# Inflammation switches the chemoattractant requirements for naive lymphocyte entry into lymph nodes

**DOI:** 10.1016/j.cell.2024.11.031

**Published:** 2024-12-20

**Authors:** Kevin Y. Chen, Marco De Giovanni, Ying Xu, Jinping An, Nikhita Kirthivasan, Erick Lu, Kan Jiang, Stephen Brooks, Serena Ranucci, Jiuling Yang, Shuto Kanameishi, Kenji Kabashima, Kevin Brulois, Michael Bscheider, Eugene C. Butcher, Jason G. Cyster

**Affiliations:** 1 Howard Hughes Medical Institute and Department of Microbiology and Immunology, University of California, San Francisco, San Francisco, CA 94143, USA; 2 Biomedical Sciences Graduate Program, University of California, San Francisco, San Francisco, CA 94143, USA; 3 Medical Scientist Training Program, University of California, San Francisco, San Francisco, CA 94143, USA; 4 Biodata Mining and Discovery Section, National Institute of Arthritis and Musculoskeletal and Skin Diseases, National Institutes of Health, Bethesda, MD 20892, USA; 5 Department of Medicine, University of California, San Francisco, San Francisco, CA 94143, USA; 6 Department of Urology, University of California, San Francisco, San Francisco, CA 94143, USA; 7 Department of Dermatology, Kyoto University Graduate School of Medicine, Kyoto 606-8507, Japan; 8 Laboratory of Immunology and Vascular Biology, Department of Pathology, Stanford University School of Medicine, Stanford, CA, USA; 9 Palo Alto Veterans Institute for Research, Palo Alto, CA, USA; 10 Present address: Division of Immunology, Transplantation, and Infectious Diseases, IRCCS Ospedale San Raffaele, Milan, Italy; 11 Present address: Gilead Sciences Inc., Foster City, CA 94404, USA; 12 Present address: Roche Pharma Research and Early Development, Roche Innovation Center, Basel 4070, Switzerland; 13 Lead contact

## Abstract

Sustained lymphocyte migration from blood into lymph nodes (LNs) is important for immune responses. The CC-chemokine receptor-7 (CCR7) ligand CCL21 is required for LN entry but is downregulated during inflammation, and it has been unclear how recruitment is maintained. Here, we show that the oxysterol biosynthetic enzyme cholesterol-25-hydroxylase (Ch25h) is upregulated in LN high endothelial venules during viral infection. Lymphocytes become dependent on oxysterols, generated through a transcellular endothelial-fibroblast metabolic pathway, and the receptor EBI2 for inflamed LN entry. Additionally, Langerhans cells are an oxysterol source. Ch25h is also expressed in inflamed peripheral endothelium, and EBI2 mediates B cell recruitment in a tumor model. Finally, we demonstrate that LN CCL19 is critical in lymphocyte recruitment during inflammation. Thus, our work explains how naive precursor trafficking is sustained in responding LNs, identifies a role for oxysterols in cell recruitment into inflamed tissues, and establishes a logic for the CCR7 two-ligand system.

## In brief

Lymph node swelling is a well-known symptom of infection. A shift in the chemoattractant code controls lymphocyte recruitment from blood into inflamed lymph nodes, allowing lymphocytes to accumulate so that even rare antigen-specific cells can encounter their activating antigen.

## Graphical abstract

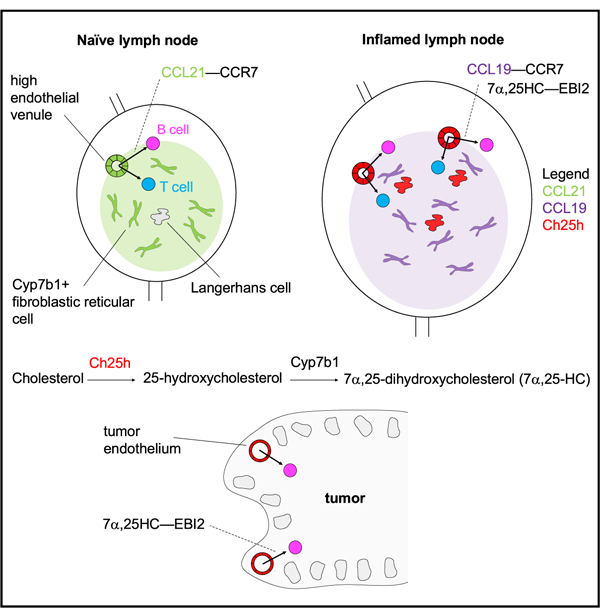

## INTRODUCTION

The human body contains ~500 lymph nodes (LNs), and circulating lymphocytes continually traffic through these immune hubs to survey for antigens that arrive via the lymph. Naive B and T lymphocytes enter LNs from the blood through high endothelial venules (HEVs).^1^ LNs draining a site of infection become inflamed and often support increased lymphocyte entry.^2–4^ By accumulating more naive cells, there is increased likelihood of a draining LN harboring lymphocytes with specificity for the foreign (or tumor) antigen and thus improved odds of mounting a protective response.^5^ While homing to LNs in homeostasis is well studied, the mechanisms supporting naive lymphocyte recruitment to inflamed LNs are less defined.

CC-chemokine receptor-7 (CCR7) is the major chemokine receptor required for naive lymphocyte entry into LNs from the blood in homeostasis, with additional contributions from CXC-chemokine receptor-4 (CXCR4) and CXCR5.^6–10^ CCR7 also plays a role in positioning T cells and dendritic cells (DCs) within the lymphoid organ.^1^ CCR7 has two ligands, CCL19 and CCL21, that are conserved across species.^1,11,12^ CCL21 has a critical role in lymphocyte recruitment to and positioning within LNs and is expressed by HEVs and T-zone fibroblastic reticular cells (FRCs).^1^ CCL19 is also highly expressed by FRCs, but its role has been less clear, as CCL19-deficient mice have intact lymphocyte and DC homing and normal LN organization.^13,14^ CCL21 is strongly downregulated in LNs after immunization or infection with bacterial or viral pathogens and in LNs draining some tumors.^15–27^ How lymphocyte recruitment is maintained under conditions of suppressed CCL21 expression during inflammation is poorly understood.

We hypothesized that alternative chemoattractant receptor-ligand systems are likely required to sustain naive lymphocyte entry from the blood into responding LNs. Here, we demonstrate that HEVs strongly express cholesterol-25-hydroxylase (Ch25h) and upregulate expression acutely during viral infection. Together with Cyp7b1, Ch25h controls the synthesis of the oxysterol 7α,25-dihydroxycholesterol (7α,25-HC), a ligand for the chemoattractant receptor EBI2 (GPR183).^28^ EBI2 is highly expressed on naive B cells and, to a lesser extent, naive CD4 and CD8 T cells. We find that the efficient entry of naive lymphocytes into inflamed LNs requires their expression of EBI2, endothelial expression of Ch25h, and stromal expression of Cyp7b1. Furthermore, we find that CCL19 is not downregulated during infection and that the CCL19-CCR7 and EBI2-oxysterol axes cooperate to support lymphocyte homing to inflamed LNs.

## RESULTS

### CCL21 downregulation and Ch25h induction in HEVs during viral infection

**Figure 1.**
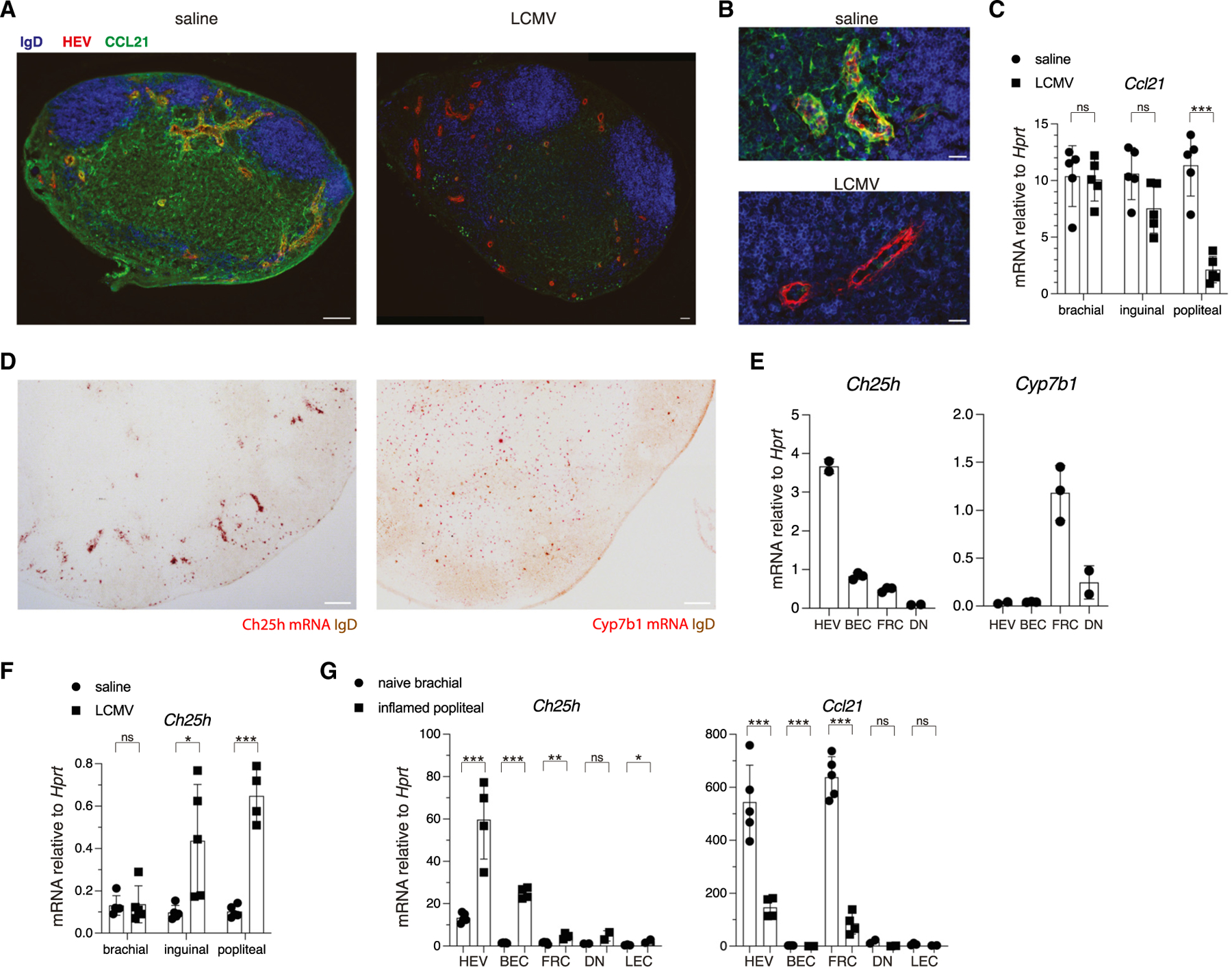
LN stroma downregulates CCL21 and upregulates Ch25h during infection (A) IFM of naive pLN (left) or pLN 24 h post-footpad LCMV infection (right). Sections stained for PNAd+ HEVs, IgD, and CCL21. Scale bars, 100 μm. (B) Zoomed-in views of HEVs in naive (top) and inflamed (bottom) LNs. Scale bars, 20 μm. (C) RT-qPCR for *Ccl21* in LNs (draining popliteal, sub-draining inguinal, or non-draining brachial) that were responding to either saline or LCMV. Each dot represents a mouse LN pair. (D) RNA-scope images of *Ch25h* and *Cyp7b1* (red) in LNs co-stained for IgD (brown). Images are representative of at least 3 mice. Scale bars, 100 μm. (E) RT-qPCR for *Ch25h* and *Cyp7b1* in sorted pLN HEVs, BECs, FRCs, or DN stromal cells. (F) RT-qPCR for *Ch25h* in LNs as in (C). (G) RT-qPCR for *Ch25h* and *Ccl21* in LN stromal cells sorted from LCMV-draining pLNs or non-draining bLNs. Each dot represents pooled sorted cells from 6 mice in (E) and (G). All statistical tests are two-tailed unpaired t tests. *p < 0.05; **p < 0.01; ***p < 0.001; ns, not significant (*p* > 0.05). Means ± standard deviation indicated in summary graphs. See also Figure S1.

Previous reports have shown that CCL21 expression decreases in various infection models, with strong downregulation after systemic infection with type-1 response-inducing pathogens after 7–8 days. To mimic a local infection scenario, we tested the effect of subcutaneous lymphocytic choriomeningitis virus (LCMV) footpad infection on CCL21 expression in the draining popliteal LN (pLN). As early as 24 h post-infection, CCL21 was strongly downregulated as detected by immunofluorescent microscopy (IFM) (Figures 1A, 1B, S1A, and S1B). In naive pLNs, CCL21 was evident on T-zone FRCs and on the abluminal and luminal sides of HEVs but nearly undetectable on HEVs and sparsely expressed on T-zone FRCs in the inflamed pLN (Figures 1A, 1B, and S1A). Transcript abundance in total LN mRNA was ~5-fold reduced (Figure 1C). CCL21 protein and mRNA remained low at day 3 post-infection (Figures S1B and S1C), and *Cxcl12* and *Cxcl13* were also repressed (Figure S1C). Because CCL21 and CXCL12 were both downregulated, and CXCL13 was slightly decreased, we wondered what other chemoattractant genes could be upregulated to fulfill the function of naive lymphocyte recruitment during inflammation. Prior bulk RNA sequencing (RNA-seq) data of LN endothelial cells showed that Ch25h, which controls the generation of 7α,25-HC (Figure S1D), was strongly expressed by HEVs.^29^ Using RNAscope, we found *Ch25h* was highly expressed in LN vasculature, as well as by interfollicular and outer T-zone cells (Figure 1D). *Cyp7b1* was not enriched in vasculature like *Ch25h* but instead expressed throughout the T-zone (Figure 1D). RT-qPCR on LN stromal cells (Figures S1E and S1F) was consistent with these findings, showing that peripheral node addressin+ (PNAd+) HEVs were enriched in *Ch25h* compared with PNAd−blood endothelial cells (BECs) and FRCs (Figure 1E). *Cyp7b1* was low in endothelium and was enriched in FRCs (Figure 1E). *CH25H* is also highly expressed in human LN HEVs and not in non-HEV endothelium (Figure S1G).

At the same 24-h time point at which CCL21 was repressed, *Ch25h* was induced ~7-fold in the draining pLN and 4-fold in the secondary draining inguinal LN (iLN) (Figure 1F). When comparing naive brachial LNs (bLNs) and draining pLNs, HEVs and BECs strongly upregulated *Ch25h*, whereas expression in FRCs, podoplanin (Pdpn) and CD31 double-negative (DN) stromal cells, and Pdpn+ CD31+ lymphatic endothelial cells (LECs) remained low (Figure 1G). Using a bioassay to measure tissue oxysterol content, 24 h inflamed LN extracts had more activity than naive LN extracts (Figure S1H), in accord with *Ch25h* expression level. Inflamed pLN HEVs and FRCs downregulated *Ccl21* 6- to 8-fold (Figure 1G), while stromal *Cyp7b1* expression did not change significantly (Figure S1I). Consistent with previous reports,^26^ inflamed FRCs downregulated *Il7* (Figure S1I). Taking these data together, the Ch25h-oxysterol-EBI2 axis stood out as a strong candidate for mediating LN lymphocyte entry during infection.

As an initial test of the ability of EBI2 to support transendothelial migration, we performed migration assays across transwell filters coated with bEnd.3 endothelial cells. B cells responded robustly to the broadly acting chemokine CXCL12 (Figure S1J). Importantly, 7α,25-HC also promoted transendothelial migration. In accord with established integrin requirements for transendothelial migration, the response in the presence but not in the absence of bEnd.3 cells was abrogated by α4 and αL blocking antibodies (Figure S1J). This integrin and EBI2-dependent migration to oxysterol was also seen for CD4 T cells (Figure S1K). Interestingly, the combination of CXCL12 with oxysterol led to greater migration compared with the sum of CXCL12 or oxysterol alone, suggestive of cooperativity between chemokine and oxysterol ligands (Figures S1J and S1K).

### EBI2 is required for naive lymphocyte entry into inflamed LNs

**Figure 2.**
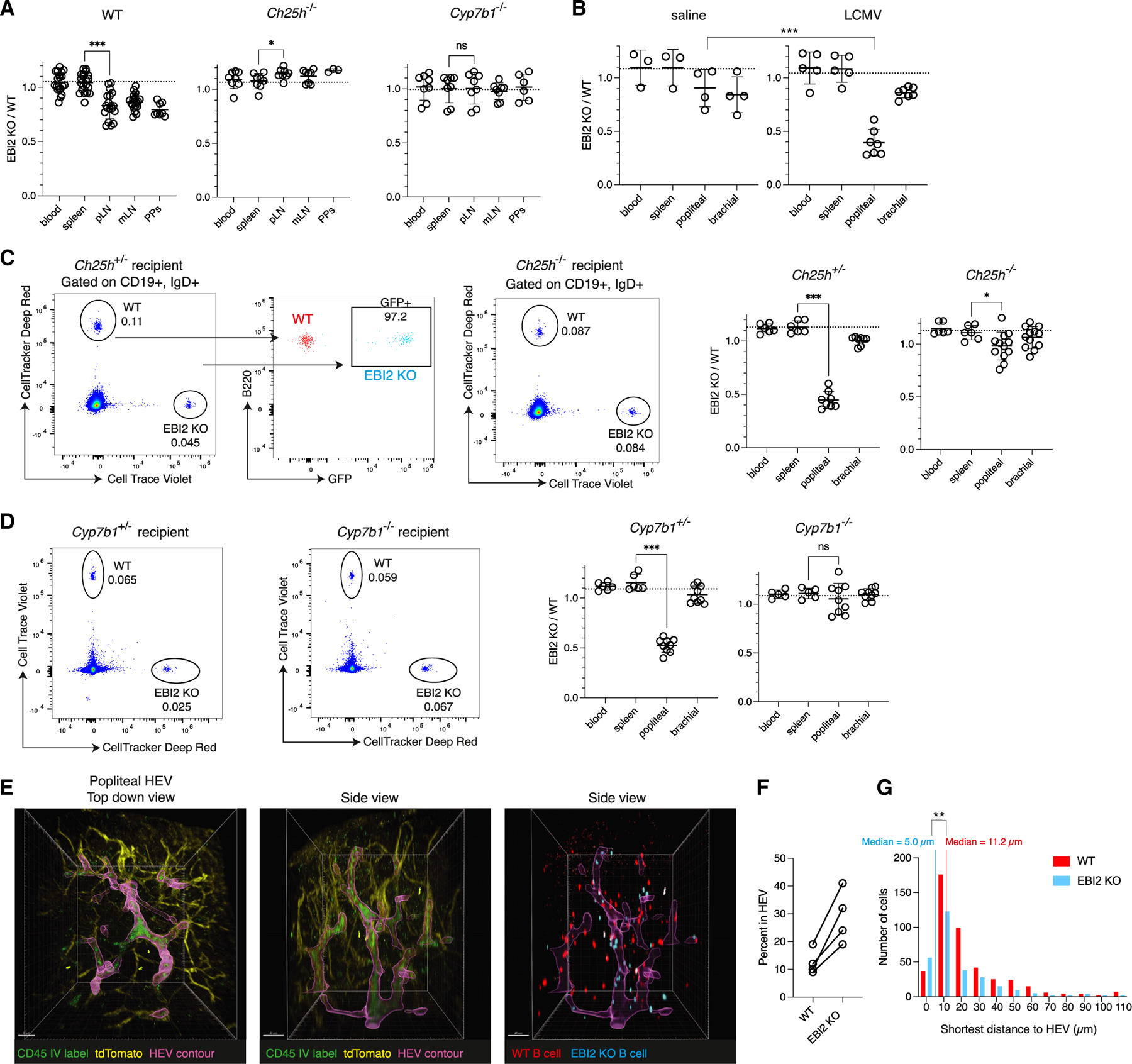
EBI2 promotes naive lymphocyte entry into homeostatic LNs and becomes essential in inflamed draining LNs (A) Quantification of transferred B cell EBI2 KO/WT ratios 90 min after co-transfer in blood, spleen, peripheral LNs, mesenteric LNs (mLNs), or Peyer’s patches (PPs) in naive WT, Ch25h−/−, or Cyp7b1−/− mice. The dashed line is the average input EBI2 KO:WT ratio. Each dot represents a mouse. (B) B cell EBI2 KO/WT ratios after co-transfer in pLNs responding to either saline or LCMV. (C) Representative flow cytometry plots of inflamed pLN from Ch25h+/— (left) or Ch25h−/− mice (right), injected 90 min prior with 50:50 EBI2 KO:WT cells. Center plot shows GFP expression in the transferred EBI2 KO B cells (cyan), which is absent in co-transferred WT cells (red). (D) Representative flow cytometry plots as in (C) with Cyp7b1+/— or Cyp7b1−/− mice. CTV and DR dyes are swapped for EBI2 KO and WT-transferred cells between (C) and (D). Summary graphs in (A)–(D) are data pooled from at least 3 experiments. Each dot represents a mouse (blood, spleen) or individual LN (popliteal, brachial) in (B)–(D). (E) Two-photon microscopy images of cleared pLNs from mTmG mice at 24 h post-footpad LCMV infection (full 3D stack in Video S1). Mice were injected with CTV+ WT B cells and DR+ EBI2 KO B cells 90 min and anti-CD45.2-AF488 (green) 2 min prior to harvest. HEV contour shown in magenta. Image is representative of 4 LNs across 2 mice and 2 independent experiments. Scale bars, 30 μm (top-down image) or 40 μm (side view images). (F) Summary quantification of E showing percent of transferred B cells within pLN HEVs. Each connected pair of dots is an LN. (G) Distribution of the shortest distance to HEV for transferred B cells, pooled from 4 LNs. Medians indicated; two-tailed unpaired t test between WT and EBI2 KO distances. Righthand edges of histogram bins listed on x axis. All statistical tests are two-tailed unpaired t tests. *p < 0.05; **p < 0.01; ***p < 0.001; ns, not significant (*p* > 0.05). Means ± standard deviation indicated in summary graphs. See also Figures S2 and S3.

To test the requirement for EBI2 in LN entry, we employed a short-term mixed adoptive transfer approach to isolate the effects of EBI2 on entry from parenchymal positioning and egress. Since Ch25h is expressed in homeostatic HEVs, we first transferred into naive mice an equal mix of EBI2−/− and wild-type (WT) splenocytes labeled with distinct dyes. At 90 min post-transfer, lymphoid organs and blood were analyzed for transferred lymphocytes. In naive peripheral LNs, as well as mesenteric LNs and Peyer’s patches, EBI2−/− B cells showed a 10%–20% deficiency compared with co-transferred WT B cells (Figure 2A). The homing effect was dependent on the EBI2 ligand 7α,25-HC, as EBI2−/− and WT B cells showed no differences in homing in Ch25h−/− or Cyp7b1−/− recipient mice (Figure 2A).

Lymphocyte homing to LNs was also tracked using an intravascular labeling approach in which the proportion of transferred cells remaining blood-exposed and thus likely in the HEV lumen 25 min post-transfer was measured using anti-CD45-PE injected 2 min before LN harvest. Consistent with less efficient transmigration into the parenchyma, a greater fraction of EBI2−/− lymphocytes remained intravascular compared with co-transferred WT cells (Figures S2A and S2B). The effect was not seen in the spleen, which does not have HEVs. This effect was also abolished in Ch25h−/− or Cyp7b1−/− mice lacking EBI2 ligand (Figures S2A and S2B).

We next assessed the contribution of EBI2 to inflamed LN homing by infecting mice with LCMV in the footpad 24 h prior to adoptive transfer. At 90 min, EBI2−/− B cells were 50%–70% less represented than co-transferred WT B cells, a significant increase in the phenotype when comparing to non-draining brachial or contralateral saline-draining pLNs (Figures 2B and S2D). Naive CD4 and CD8 T cells also displayed EBI2 homing dependency to inflamed LNs, though to a lesser degree, reflecting their lower EBI2 and higher CCR7 expression (Figures S2C and S2D). When comparing naive to inflamed LNs, B cells increased in entry, and CD4 and CD8 T cell entry were maintained at similar levels (Figure S2E). EBI2−/− B cells were unable to increase in entry, and EBI2−/− T cells could not maintain their homeostatic homing efficiency (Figure S2E). As in naive mice, the homing effects in inflamed LNs were Ch25h and Cyp7b1 dependent (Figures 2C and 2D) and dye-labeling independent (Figures 2C and 2D). Compared with WT recipients, draining pLNs from Ch25h−/− and Cyp7b1−/− mice showed less recruitment of WT-transferred B cells (Figure S2F). In comparison, EBI2−/− B cells homed at similar lower levels in both inflamed WT and Ch25h−/− or Cyp7b1−/− pLNs, consistent with their inability to sense oxysterols (Figure S2F). Furthermore, endogenous numbers of B cells were lower in inflamed pLNs of Ch25h−/− or Cyp7b1−/− compared with littermate controls but not in non-draining bLNs (Figure S2G).

We were interested to know if the increased reliance on EBI2 ligands extended beyond the acute phase of infection and found that at days 5 and 7 post-LCMV footpad infection, EBI2-deficient B cells were still strongly deficient in homing (Figure S2H). Although LCMV footpad infections are cleared by days 8–10,^30,31^ *Ccl21* expression was still recovering at day 14 in the pLN and reached 80% of baseline expression at day 36 (Figure S2I). Importantly, naive B cell entry no longer displayed increased EBI2 dependence in this resolution phase (Figure S2J). Since the CD8 T cell response to LCMV can cause LN immunopathology,^32^ potentially leading to B cell entry no longer being increased at day 5 post-infection (Figure S2H), we examined entry at day 5 post intranasal influenza infection. In this setting, increased B cell entry into the draining mediastinal LN was also reliant on EBI2 (Figure S2K). Additionally, we titrated down the infection dose by 100-fold in the LCMV footpad setting and found that the magnitude of the EBI2−/− B cell homing defect at day 3 post-infection was unchanged (Figure S2L). Finally, to test whether alterations in LN egress might contribute to the short-term transfer results, we blocked LN entry after the 90 min transfer using intravenous (IV) α4 and αL integrin neutralizing antibodies. The extent of decay of transferred EBI2−/− and WT B cells in the inflamed pLNs after 18 h was similar (Figure S2M), suggesting no egress phenotype.

To analyze EBI2−/− entry effects by imaging, a 90-min mixed transfer of dye-labeled B cells was performed in mTmG mice, which express tdTomato highly in endothelium and thus provide a fluorescent readout of vasculature. 2 min before harvest, anti-CD45-FITC (fluorescein isothiocyanate) was injected IV to label stretches of high-density lymphocytes in LN vessels, thus highlighting HEVs. LNs were then cleared and imaged via two-photon microscopy to obtain three-dimensional views of HEV networks (Figure 2E; Video S1). In inflamed pLNs, 24 h post-infection, EBI2−/− B cells remained in HEVs at greater proportions than WT co-transferred B cells (Figure 2F). This effect was present but less pronounced in naive bLNs (Figures S3A and S3B; Video S2). To assess B cell migration away from HEVs, regions centered on HEVs were obtained, and the shortest distance of transferred B cells to HEVs was measured. The median distance to HEVs for WT B cells was ~2-fold greater than that for EBI2−/− B cells (Figure 2G), an effect that was again seen to a lesser degree in the uninflamed bLN (Figure S3C). Furthermore, in agreement with flow cytometry analyses, the ratio of EBI2−/−:WT B cells was ~50% reduced compared with blood in pLNs and ~20% reduced in bLNs (Figure S3D).

To confirm the entry requirement for EBI2 was not specific to short-term transfer systems, we footpad-infected mixed chimeras and assessed EBI2−/−:WT representation 72 h post-infection. EBI2−/− lymphocytes were less represented than WT lymphocytes in draining pLNs compared with spleen and non-draining bLNs (Figure S3E). Furthermore, to ensure the entry effect was not specific to anatomic draining location, mice were infected with LCMV in the tail base to target the iLNs. EBI2−/− B cells displayed significant homing defects in inflamed iLNs (Figures S3F and S3G). CCL21 was also downregulated in inflamed iLNs, but there was greater heterogeneity in downregulation across the two lobes and HEVs of the iLN (Figure S3H). This could be due to additional sites of drainage not targeted with a tail base subcutaneous injection. Overall, these results suggest that inflammation drives an increased dependency on EBI2 for naive lymphocyte entry into the draining LN.

### CCL21 downregulation increases EBI2-dependency of inflamed LN entry

**Figure 3.**
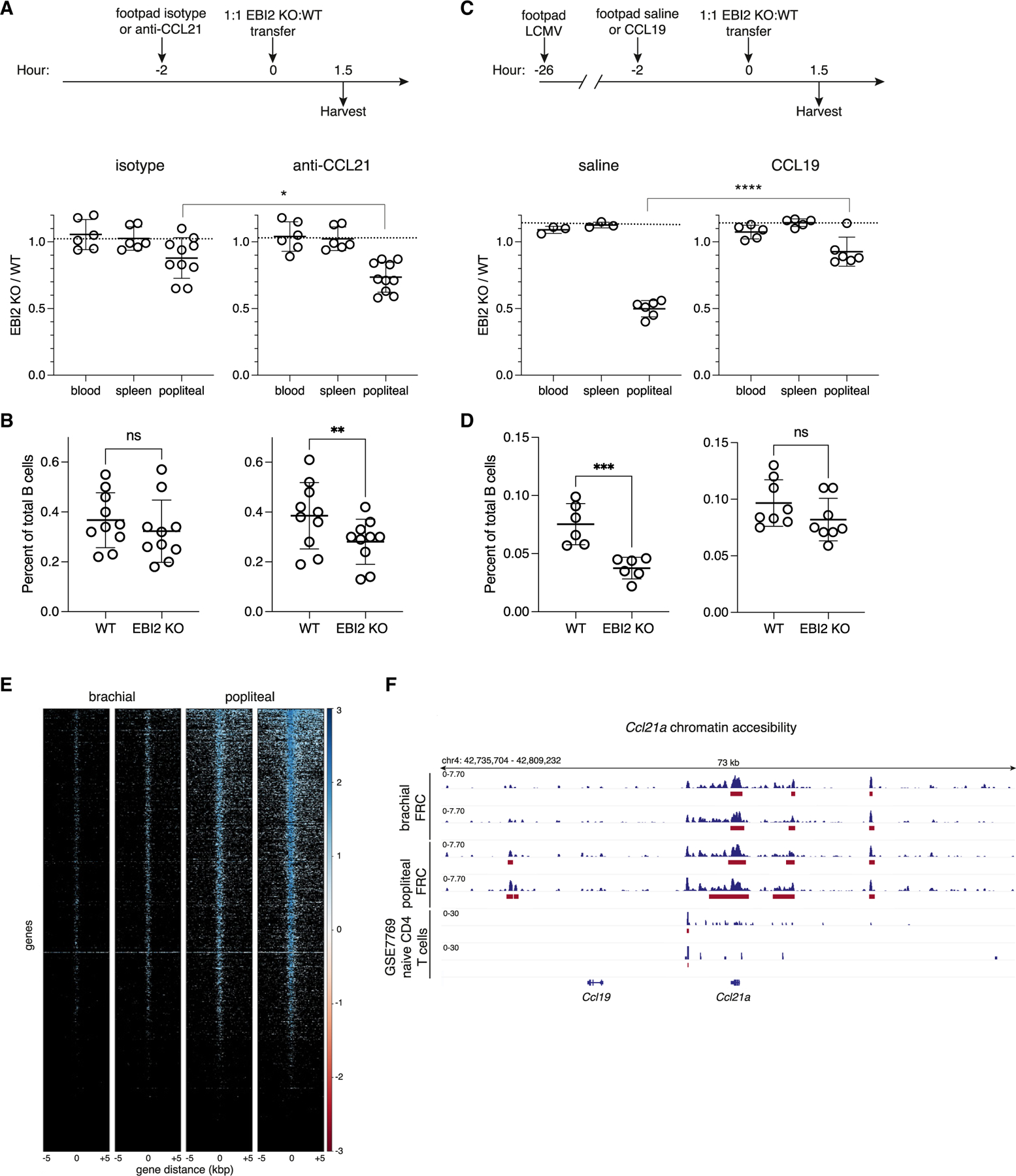
CCL21 blockade increases the EBI2 requirement for LN homing, while CCL19 delivery reverses it in inflamed LNs (A) Schematic of experiment (top). Quantification of EBI2 KO:WT B cell ratios 90 min post-transfer in naive pLNs. (B) Percent of total B cells that are transferred WT or EBI2 KO B cells in naive pLNs. (C) Schematic of experiment (top). Quantification of transferred EBI2 KO:WT B cell ratios post-transfer in inflamed pLNs. The dashed line is the average input ratio from 3 experiments in (A) and (C). (D) Percent of total B cells that are transferred WT or EBI2 KO B cells in inflamed pLNs. Each dot represents a mouse (blood, spleen) or individual LN (popliteal) in (A)–(D). (E) ATAC-seq heatmap showing chromatin accessible regions around transcription start site (TSS), 5 kb flanking on both sides in inflamed pLN and naive bLN FRC. (F) ATAC-seq reads coverage for *Ccl21a*. Top two tracks are non-draining bLN FRC duplicates, and middle two tracks are LCMV-inflamed pLN FRC duplicates. Bottom two tracks are CD4 T cell duplicates from GEO: GSE77695.^33^ Red bars indicate ATAC-seq peaks. All statistical tests are two-tailed unpaired t tests. *p < 0.05; **p < 0.01; ***p < 0.001; ****p < 0.0001; ns, not significant (*p* > 0.05). Means ± standard deviation indicated in summary graphs. See also Figures S3 and S4.

We next asked whether CCL21 suppression is sufficient to account for the increased role of EBI2 in LN entry. To test this, we injected CCL21 neutralizing or control antibody into the footpads of naive mice (Figure 3A). 2 h later, we performed the 90 min mixed transfer. In naive LNs draining anti-CCL21, EBI2−/− B cells showed an increased deficiency compared with LNs draining isotype control (Figures 3A and 3B). The increase in EBI2’s homing role was not as large as occurring after infection, perhaps due to incomplete CCL21 blocking and lack of Ch25h upregulation.

Inversely, we attempted to rescue the EBI2−/− lymphocyte homing to inflamed LNs by providing CCR7 ligand. We infected with LCMV 24 h prior to injecting the footpads with recombinant CCL21, CCL19, or saline (Figures 3C and S3I). 2 h later, EBI2−/− and WT splenocytes were transferred. CCL21 injection was ineffective in restoring EBI2−/− B cell homing (Figure S3I). However, CCL21 is highly positively charged, and its strong binding to glycosaminoglycans^34,35^ may prevent it from traveling efficiently from a site of inflammation. The second CCR7 ligand, CCL19, is less positively charged and has been shown to reach draining LNs following subcutaneous injection,^35^ and we therefore tested it as a surrogate for CCL21. We stained for CCL19 in naive and inflamed LNs and found localization to T-zone FRCs, which was not present in CCL19−/− control sections (Figure S3J). Injection of CCL19 into inflamed footpads markedly increased the CCL19 that could be detected around the LN sinuses and medulla (Figure S3K). While there is evidence that CCL19 can be transcytosed onto HEVs,^36^ endogenous HEV-associated CCL19 was difficult to detect (Figure S3L). However, after exogenous delivery, CCL19 was detectable on HEVs (Figure S3L). When we performed short-term transfers, pLNs from mice given CCL19 showed EBI2−/− B cells homing at nearly the same level as WT-transferred B cells (Figures 3C and 3D), thus reversing the effect of infection-induced CCL21 downregulation. Similar effects were observed for CD4 and CD8 T cells (Figure S3M). Taken together, these results suggest that CCL21 suppression during infection leads to an increased dependence on oxysterol-EBI2 for lymphocyte homing to draining LNs.

As a further test of the relation between CCL21 repression and oxysterol dependence, we investigated LN homing of antigen-experienced lymphocytes, some subsets of which can express CCR7 and retain the ability to recirculate through lymphoid organs. Of note, CD8 T central memory (Tcm) cells maintain L-selectin and CCR7 expression but are also enriched for EBI2 compared with naive or L-selectin-negative effector cells (Figure S4A).^37^ However, CD8 memory and effector cells also express inflammatory chemokine receptors, and the induction of inflammatory chemokines may promote their entry to LNs independently of CCL21 or oxysterols. CXCL9, CXCL10, and CXCL16 were strongly upregulated in draining pLNs at day 1 post-LCMV infection, but this induction dampened by day 3 (Figure S4B). Since CCL21 repression persists throughout LCMV infection (Figure S2I),^17^ we reasoned that CD8 Tcm might experience a switch toward oxysterol-dependent homing at later time points. While CD8 Tcm showed no homing requirement for EBI2 at day 1 post-infection, there was a ~50% deficiency in EBI2−/− CD8 Tcm entry at day 5 (Figures S4C and S4D). Another CCR7- and EBI2-expressing subset, PD1+ SLAMF6+ TIM3− T-precursor exhausted cells (Figure S4E),^38^ also displayed LN entry dependence on EBI2 at day 5 (Figures S4F and S4G). As a complementary assessment, we analyzed mixed chimeras at day 8 post-footpad LCMV infection. Ratios of EBI2 knockout (KO) to WT cells for both CD8 subsets were reduced in pLNs compared with spleen (Figure S4H). WT:WT chimeras showed no difference. The mixed chimeras potentially corroborate the short-term entry experiments, although we cannot exclude parenchymal positioning effects on T cell maintenance and exhaustion.^39^ Overall, these data suggest that the temporal interplay between CCL21 and oxysterols impacts lymphoid homing of memory/progenitor populations as well.

We also examined other inflammation settings to better understand the signals mediating EBI2 dependency for LN homing. Increased EBI2 dependency was observed at both 24 h post-vesicular stomatitis virus (VSV) infection and day 4 post-complete Freund’s adjuvant (CFA) immunization (Figures S4I–S4K). CCL21 downregulation was observed by IFM in CFA-draining pLNs (Figure S4L) and by RT-qPCR for both VSV and CFA (Figures S4M and S4N). Similar to LCMV, Ch25h induction faded by days 3–4 for VSV and CFA. However, EBI2 homing dependency persisted at these time points (Figures S2D and S4K), indicating that Ch25h upregulation likely promotes but is not required for the switch toward oxysterol-driven entry. In line with this, the EBI2−/− homing defect at 24 h post-LCMV infection was slightly diminished but still robust in interferon alpha and beta receptor subunit 1 (IFNAR1)−/− mice (Figure S4O), whose pLNs showed blunted *Ch25h* induction (Figure S4P). *Ccl21* downregulation, however, was equally strong in IFNAR1−/− and WT mice (Figure S4P). In contrast to IFNAR1−/− mice, IFNγ−/− and IFNγR1−/− mice showed no decrease in the EBI2−/− homing deficiency to 24 h LCMV-inflamed LNs (Figure S4Q), consistent with Ch25h induction being largely interferon (IFN)-α/β driven. *Ccl21* downregulation was also marked in IFNγR1−/− pLNs (Figure S4R).

To investigate the mechanism of CCL21 suppression in inflamed LNs, we performed assay for transposase-accessible chromatin with sequencing (ATAC-seq) on sorted FRCs from naive and inflamed LNs. However, CCL21 is encoded in a ~1,000 kb repetitive region on chromosome 4,^40^ which includes *Ccl19* and *Ccl19*-pseudogenes, two *Il11Ra* genes, two *Ccl27* genes, and three *Ccl21* genes, *Ccl21a*, *Ccl21b*, and *Ccl21c*, with Ccl21a being the gene that is active in LNs.^12,13^ This region has not been annotated in ENCODE or other lists, and published ATAC-seq analyses report no mapped reads (Figure S4S). Using a pipeline that reports uniquely mapped paired reads, we obtained coverage for this region (Figure S4S). As a control, *Il7* was open and accessible in this FRC dataset but not in published T cell datasets (Figure S4T). Inversely, *Il7r* was accessible in CD4 T cells but not FRCs (Figure S4T). Globally, inflamed LN FRC genes were more accessible than naive LN FRC genes (Figures 3E and S4U). This included *Ch25h* and additional IFN-stimulated genes (ISGs), such as *Ifitm3* (Figure S4T). Notably, the *Ccl21a* and *Ccl19* loci were accessible in FRCs and not in T cells, but accessibility was not significantly different between inflamed and naive LNs (Figure 3F). This suggests mechanisms other than decreased chromatin accessibility lead to CCL21 suppression. When performing gene ontology and transcription factor regulation analysis on the most differentially accessible genes between inflamed and naive FRCs, NFkb1 emerged, as expected, as well as other factors such as SMAD3/4 (Figures S4V and S4W). In support of the latter finding, *Tgfβr1*, *Tgfβ3*, and *Tgfβr3* were increased in accessibility (Figure S4X). RNA-seq from LN fibroblasts of mice systemically infected with LCMV-clone13^41^ also showed increased expression of *Tgfβr1*, *Tgfβ3*, and *Tgfβr3*, consistent with the ATAC-seq data (Figures S4X and S4Y). Transforming growth factor β (TGF-*β*) signaling thus may be increased in inflamed LN fibroblasts, and this could provide feedback for FRC antiviral responses or potentially mediate CCL21 downregulation.

### Endothelial Ch25h is required for naive lymphocyte recruitment to inflamed LNs

To investigate the cell types sourcing Ch25h for EBI2-mediated LN entry, we started with assessing the endothelial-specific contribution since Ch25h is highly expressed in HEVs. We used a tamoxifen-inducible Cdh5-CreERT2 (iCdh5) model with floxed Ch25h alleles (Ch25h^ΔBEC^) to delete Ch25h in endothelium specifically, without effects on hematopoietic cell types that have been reported for constitutive endothelial Cre lines. By crossing the iCdh5 mice with mTmG reporter mice, we found that Cdh5-CreERT2 is highly efficient in LN HEVs and BECs after tamoxifen treatment (Figures S5A and S5B). There was little Cre activity in FRCs (Figures S5A and S5B). Cdh5-CreERT2 can have activity in lymphatic endothelium as well,^42^ but we found little *Ch25h* expression in LN lymphatics (Figure 1G).

**Figure 4.**
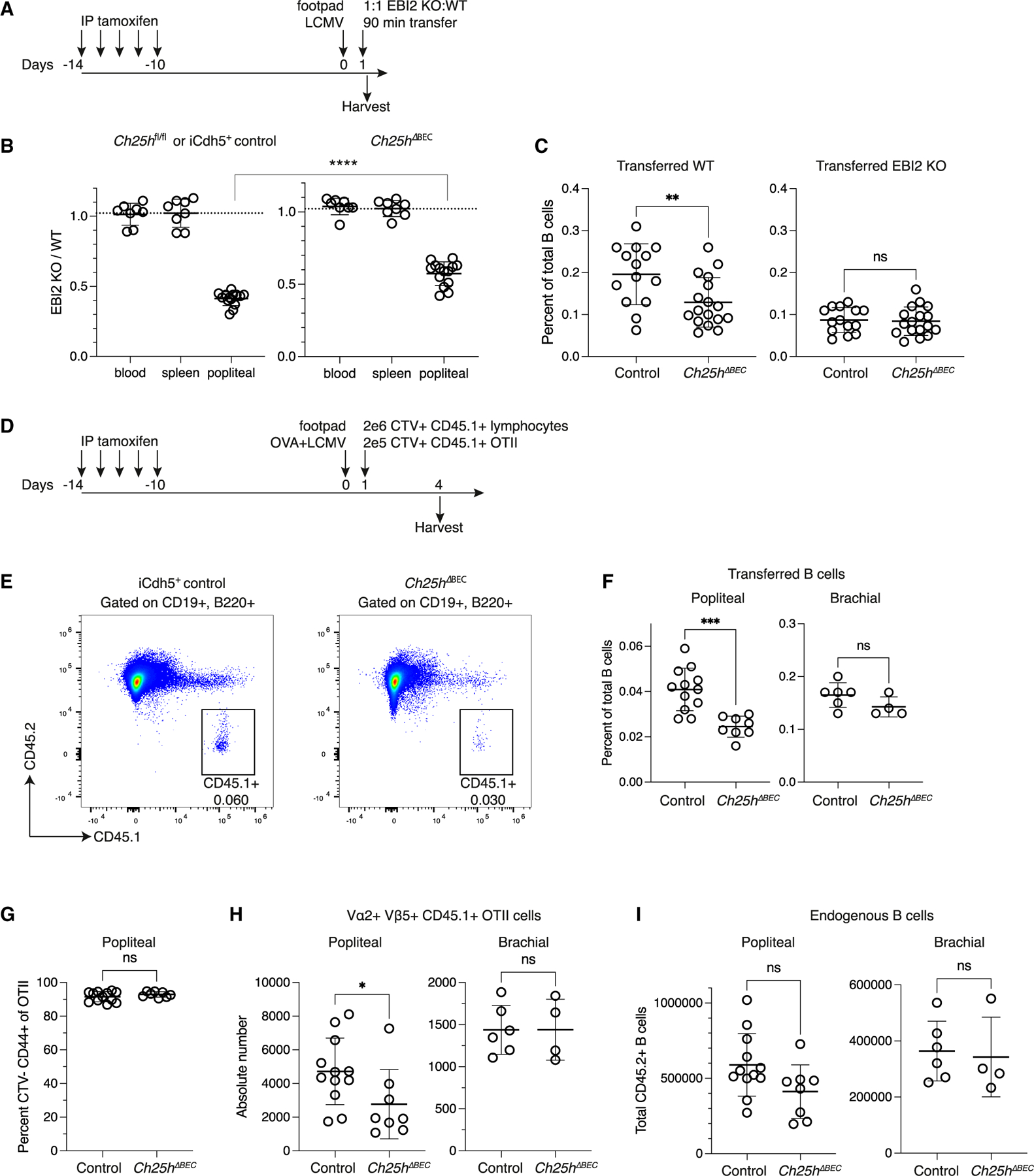
Endothelial Ch25h is necessary for optimal lymphocyte recruitment and antigen-specific T cell response in inflamed draining LNs (A) Schematic of experiment. (B) Quantification of EBI2 KO:WT B cells after co-transfer in LCMV-infected control (iCdh5+ or Ch25h^fl/fl^) and Ch25h^ΔBEC^ mice. The dashed line is the average input ratio from 5 experiments. (C) Transferred B cell percent of total B cells in control and Ch25h^ΔBEC^ pLNs from (B). (D) Schematic of experiment. (E) Representative flow cytometry plot of CD45.1+ transferred B cells in inflamed pLNs in control (iCdh5+ or Ch25h^fl/fl^) and Ch25h^ΔBEC^ mice. (F) Quantification of transferred B cells in (E), 3 days after transfer (4 days after LCMV+OVA footpad injection). (G) Percent of transferred OTII CD4+ T cells that have diluted CTV and are CD44+ in inflamed draining pLNs of control and Ch25h^ΔBEC^ mice. (H) Absolute numbers of transferred OTII cells in draining pLNs and non-draining bLNs of control and Ch25h^ΔBEC^ mice. (I) Total host endogenous B cells in draining pLNs and non-draining bLNs of control and Ch25h^ΔBEC^ mice, 4 days after LCMV+OVA footpad injection. Data pooled from 2 independent experiments in (F)–(I). Each dot represents a mouse (blood, spleen) or individual LN (popliteal, brachial) in (B), (C), and (F)–(I). All statistical tests are two-tailed unpaired t tests. *p < 0.05; **p < 0.01; ***p < 0.001; ****p < 0.0001; ns, not significant (*p* > 0.05). Means ± standard deviation indicated in summary graphs. See also Figure S5.

After tamoxifen treatment, control and Ch25h^ΔBEC^ mice were infected with LCMV 24 h before mixed EBI2−/−:WT lymphocyte transfer (Figure 4A). Compared with tamoxifen-treated controls, ratios of transferred EBI2−/− to WT B cells in Ch25h^ΔBEC^ pLNs were closer to that of the spleen and blood, indicating a reduction of EBI2 dependence due to decreased ligand (Figure 4B). Similar to full Ch25h−/−, transferred WT B cells also homed less efficiently to Ch25h^ΔBEC^ inflamed LNs (Figure 4C). In comparison, EBI2−/− B cells homed to similar extents in control and Ch25h^ΔBEC^ mice (Figure 4C). However, the Ch25h^ΔBEC^ effects were partial, and this could not be explained by inefficient Cre activity (Figures S5A and S5B).

To investigate longer-term effects of endothelial Ch25h deficiency, including effects on the CD4 T cell response to antigen, we transferred CTV-labeled CD45.1+ WT cells and ovalbumin (OVA)-specific OTII CD4 T cells into control and Ch25h^ΔBEC^ mice that had been immunized with LCMV and OVA and analyzed the recipients 3 days later (Figure 4D). B cells homed less efficiently to the draining pLNs of Ch25h^ΔBEC^ mice but were at similar frequencies between control and Ch25h^ΔBEC^ mice in the non-draining bLNs (Figures 4E and 4F). The OTII cells completely diluted CTV due to proliferation in the draining pLN, with some proliferated OTII cells appearing in the bLN, likely due to recirculation (Figures 4G and S5C). However, there were fewer OTII cells in the draining pLNs of Ch25h^ΔBEC^ mice compared with WT mice, whereas bLNs showed no difference (Figure 4H). Polyclonal CD4 and CD8 T cells injected with the OTII cells also homed less efficiently to draining pLNs of Ch25h^ΔBEC^ mice, but like B cells, they homed with similar efficiencies to non-draining bLNs (Figure S5D). Endogenous lymphocyte numbers followed similar trends, with total B and T cells being significantly reduced, or trending toward significance, in Ch25h^ΔBEC^ pLNs but not bLNs (Figures 4I and S5E). Overall, these data suggest that endothelial Ch25h plays an important role in supporting optimal lymphocyte entry and LN enlargement during inflammation, with the reduced naive precursor T cell recruitment in the absence of endothelial Ch25h leading to a diminished CD4 T cell response.

### Langerhans cells promote lymphocyte homing to inflamed LNs

**Figure 5.**
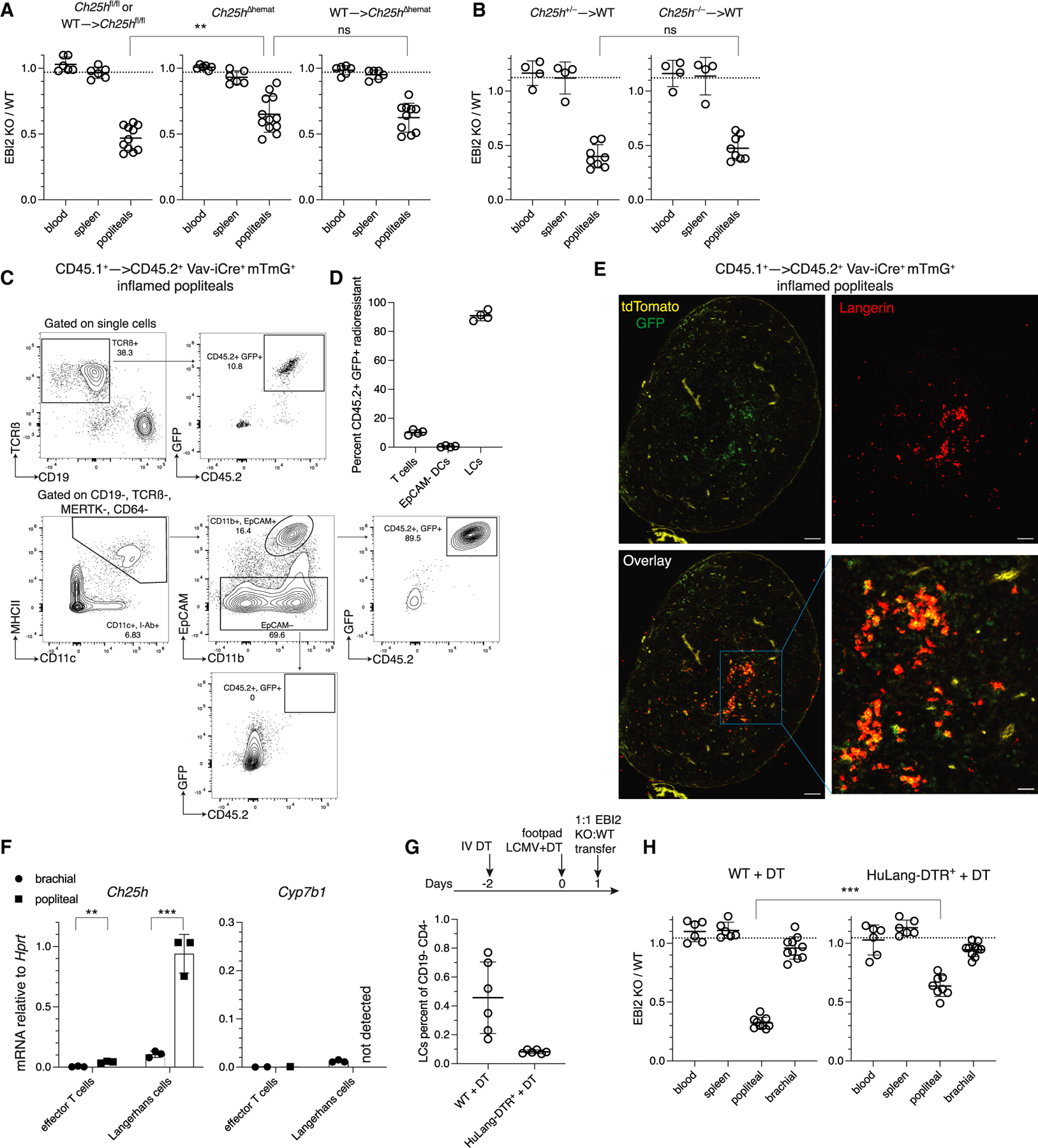
Radioresistant Langerhans cells contribute to recruiting naive lymphocytes into the draining LN (A) Quantification of EBI2 KO:WT B cell ratios after co-transfer in control (Ch25h^fl/fl^ mice or WT→Ch25h^fl/fl^ chimeric mice), Ch25h^Δhemat^, or WT→Ch25h^Δhemat^ chimeric mice, post LCMV infection. (B) Quantification of EBI2 KO:WT B cell ratios after co-transfer in Ch25h+/−→WT or Ch25h−/−→WT chimeras, post LCMV infection. The dashed line is the average input ratio from 3 experiments in (A) and (B). (C and D) Representative flow cytometry plots of radioresistance in T cells, EpCAM+ CD11b+ Langerhans cells (LCs), and EpCAM− DCs in CD45.1+ WT→Vav-iCre+ mTmG+ chimeras. Each dot represents pooled pLNs from a mouse in (D). (E) IFM of LCMV-inflamed pLN in WT→Vav-iCre+ mTmG+ chimeras, stained with anti-Langerin for LCs, with endogenous tdTomato and GFP signal. Sections representative of 2 mice. Scale bars, 100 or 30 μm for inset. (F) RT-qPCR for *Ch25h* and *Cyp7b1* in LN effector CD44+ CD62L− CD4 T cells and LCs sorted from either LCMV-draining pLNs or non-draining bLNs. Each dot represents pooled sorted cells from 3–5 mice. *Cyp7b1* undetectable in some samples. (G) Schematic of experiment (top) and efficiency of LC ablation in HuLang-DTR+ mice. Each dot represents pooled iLNs from a mouse. (H) Quantification of transferred B cell EBI2 KO:WT ratios after co-transfer in DT-treated WT or HuLang-DTR+ mice, post LCMV infection. The dashed line is the average input ratio from 3 experiments. Each dot represents a mouse (blood, spleen) or individual LN (popliteal, brachial) in (A), (B), and (H). All statistical tests are two-tailed unpaired t tests. **p < 0.01; ***p < 0.001; ns, not significant (*p* > 0.05). Means ± standard deviation indicated in summary graphs. See also Figures S5 and S6.

Given the partial phenotype of endothelial-specific Ch25h deficiency, we asked what other sources of Ch25h contribute to inflamed LN homing. Ch25h is upregulated in myeloid cells by IFN-α/β, and we therefore tested the hematopoietic compartment for any contribution to EBI2-dependent lymphocyte entry. Using Vav-iCre+ Ch25h^f/f^ mice (Ch25h^Δhemat^) to delete Ch25h in all hematopoietic cells, we found a partial decrease in EBI2-dependent B cell homing to inflamed pLNs, similar to the magnitude of the endothelial Ch25h^ΔBEC^ phenotype (Figure 5A).

We next tested whether transferring WT bone marrow (BM) into irradiated Ch25h^Δhemat^ mice would restore oxysterol generation and EBI2 homing dependency, but surprisingly, we observed that WT→Ch25h^Δhemat^ chimeras showed little change in EBI2−/− to WT B cell recruitment compared with non-chimeric Ch25h^Δhemat^ mice (Figure 5A). This indicated that a radioresistant, Vav-iCre active, hematopoietic cell type was contributing Ch25h to EBI2-dependent LN homing.

To confirm this finding, we transferred Ch25h+/− or −/− BM into WT mice to test whether the loss of Ch25h in radiosensitive hematopoietic cells would decrease EBI2 dependency in homing. Consistent with the Ch25h^Δhemat^ results, Ch25h+/−→WT and Ch25h−/−→WT chimeras were similar in their preference to recruit WT-transferred B cells over EBI2−/− B cells, indicating a significant contribution of Ch25h by radioresistant cells (Figure 5B). The EBI2−/− homing defect for CD4 and CD8 T cells was also unaffected by Ch25h−/− BM reconstitution (Figure S5F). This indicated that Ch25h expression in radiosensitive cells was not necessary to provide ligand for EBI2-dependent lymphocyte homing to the inflamed LN.

To identify radioresistant hematopoietic cell types that might contribute Ch25h to homing, we generated CD45.1+ WT→ CD45.2+ Vav-iCre+ mTmG+ chimeras. In these mice, all non-hematopoietic, radioresistant cells express tdTomato while hematopoietic radioresistant cells express GFP (Figure 5C). This analysis established that Langerhans cells (LCs) were the major radioresistant hematopoietic cell type in inflamed pLNs and that 80%–90% of the LCs were GFP+ radioresistant cells (Figure 5D). The high radioresistance of LCs is in accord with previous studies.^43^ CD44+ CD62Llow effector T cells exhibited ~10% radioresistance, and EpCAM− DCs were completely replaced by donor BM-derived cells (Figure 5D). By microscopy, inflamed pLNs displayed GFP+ radiation-resistant cells in the T-zone that were mostly Langerin+, with some scattered GFP+ cells in between that were likely T cells (Figures 5E, S5G, and S5H). We also analyzed the radioresistance of all CD64+ macrophages in the inflamed pLN and found 97%–99% replacement by donor BM-derived cells (Figures S5I–S5K). Finally, due to some Vav-Cre lines being active in endothelium, we confirmed the Vav-iCre line was inactive in stromal cells in inflamed pLNs (Figure S5L).

We tested whether Ch25h was expressed in radioresistant LCs or effector CD4 T cells (Figure S6A). Both expressed little to no Ch25h in naive non-draining bLNs (Figure 5F). LCs markedly upregulated Ch25h in inflamed pLNs (Figure 5F) but not yet in the footpad skin at day 1 after LCMV challenge (Figure S6B). LCs take several days to reach draining LNs from inflamed skin.^44^ We suggest that footpad LCMV challenge leads to acute upregulation of Ch25h on LCs already present in LNs. Cyp7b1 was lowly expressed in T cells and LCs from naive and inflamed LNs (Figure 5F), suggesting that T-zone FRCs surrounding the LCs would be required for LC-derived EBI2 ligand generation.

To investigate the role of DCs in inflamed LN lymphocyte recruitment, we generated Zbtb46-DTR+→WT chimeras and WT→WT control chimeras. Zbtb46 is expressed in all conventional dendritic cells (cDCs) and LCs,^45^ but because LCs are mostly radioresistant, only cDCs are depleted after diphtheria toxin (DT) treatment in Zbtb46-DTR+→WT chimeras (Figure S6C). CD11b^hi^ monocyte-derived DCs do not express Zbtb46 and remain after DT treatment (Figure S6C). In these chimeras, there was a modest, but not statistically significant, decrease in the EBI2−/− homing phenotype (Figure S6D), indicating that cDCs were not a major contributor of Ch25h for homing. To further investigate the role of LCs, we employed the HuLangDTR+ model, which uses a human Langerin-DTR construct to direct DTR expression in LCs but not Langerin+ dermal DCs.^46^ After DT treatment of HuLangDTR+ mice, there was significant, but not complete, LC depletion and no depletion of dermal DCs or resident CD8+ DCs (Figures 5G and S6E–S6G). In the HuLangDTR+ mice, there was a decrease in the preference for WT over EBI2−/− B cells in LN entry, similar in magnitude to the partial phenotype of Ch25h^ΔBEC^ or Ch25h^Δhemat^ mice (Figure 5H). By contrast, there was no effect of LC depletion on EBI2-dependent homing to naive bLNs, consistent with the low Ch25h expression in naive LCs. These data provide evidence that LCs contribute to EBI2 ligand generation and lymphocyte recruitment to inflamed LNs.

### EBI2 promotes B cell homing in a solid tumor model

**Figure 6.**
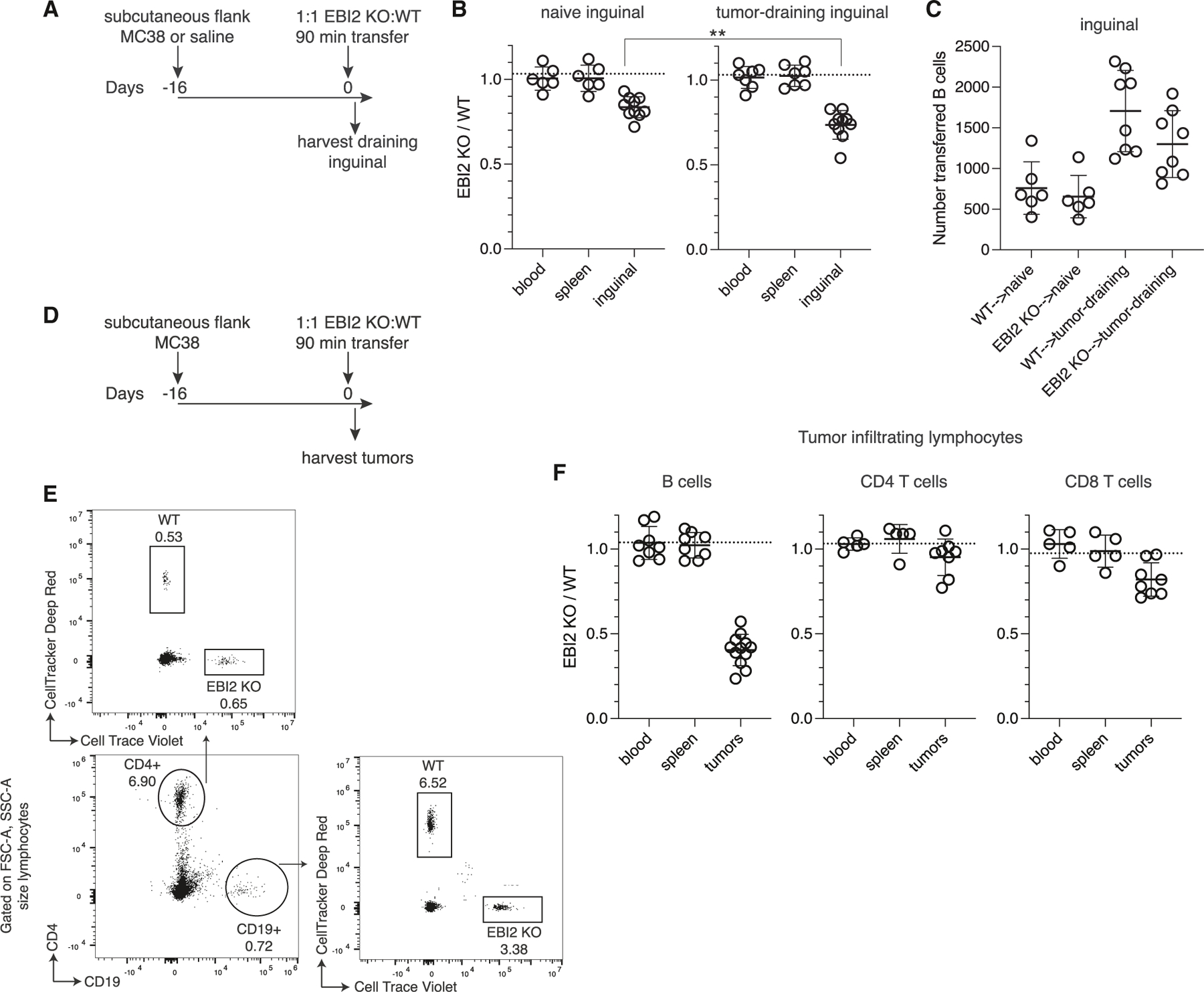
EBI2 is required for naive B cell homing to tdLNs and solid tumors (A) Schematic of experiment. (B) Quantification of EBI2 KO:WT B cell ratios after co-transfer in saline-injected or MC38 tumor flank-implanted mice. The dashed line is the average input ratio from 5 experiments. Statistical test is two-tailed unpaired t test. **p < 0.01. (C) Absolute numbers of transferred B cells in naive or tumor-draining iLNs. Data pooled from 3 experiments. (D) Schematic of experiment. (E) Representative flow cytometry plots of transferred EBI2 KO and WT B cells and CD4 T cells that have infiltrated the solid tumor, 90 min post co-transfer. (F) Quantification of transferred B, CD4, and CD8 T cell EBI2 KO:WT ratios after co-transfer in tumor-implanted mice. The dashed line is the average input ratio from 3 to 5 experiments. Each dot represents a mouse (blood, spleen) or individual tumors or iLNs in (B), (C), and (F). Tumors implanted bilaterally in some mice. Means ± standard deviation indicated in summary graphs. See also Figure S7.

Because tumor-draining LNs (tdLNs) have been reported to downregulate CCL21 in some mouse model and human studies,^15,19,20,24,25,27^ we tested whether iLNs responding to subcutaneous MC38 tumors would exhibit increased EBI2 dependency for naive lymphocyte recruitment (Figure 6A). EBI2−/− B cells were slightly but significantly more deficient in homing to tdLNs compared with naive LNs (Figure 6B). B cell entry into tdLNs was increased compared with naive LNs (Figure 6C), and EBI2−/− B cells could not enter at the same elevated level as WT B cells. The increased EBI2 dependency was consistent with moderate CCL21 downregulation in tdLNs compared with contralateral naive LNs (Figure S7A). Suppression of *Ifnb1* and *Ifng* also coincided with a lack of Ch25h induction in tdLNs. Overall, tdLNs in the MC38 model somewhat mimic the environment of a virally infected LN, thus increasing the requirement of EBI2 in ongoing B cell recruitment to tdLNs.

Since inflamed LNs lose CCL21 while increasing oxysterol-synthesizing capacity, we also sought analogous chemoattractant environments in peripheral sites of inflammation. In a subcutaneously injected fibrosarcoma tumor model,^47^ *Ch25h* was present in tumor-associated endothelium while *Ccl21* was absent (Figure S7B). In another peripheral site, the skin, *CH25H* was detected in *ACKR1*+ post-capillary endothelial cells from skin biopsies of patients with chronic atopic dermatitis (Figure S7C).^48^ *CCL21* and *CCL19* were not detected. These data suggested that chemotactic oxysterols may function in EBI2+ cell recruitment to inflamed nonlymphoid tissues.

To test for EBI2 function in naive lymphocyte homing to peripheral sites, we employed the subcutaneous MC38 tumor model since it is a “hot” tumor with increased immune cell infiltration compared with other murine tumor lines.^49^ At 16 days post implantation, EBI2−/− and WT lymphocytes were transferred IV, and tumors were analyzed 90 min later to assess entry-specific effects (Figure 6D). There was a 50%–70% deficiency of EBI2−/− B cells in the tumor (Figures 6E and 6F), similar to the homing defect in inflamed LNs. Swapping the dyes between EBI2−/− and WT lymphocytes did not affect the tumor entry defect (Figures S7D and S7E). The entry phenotype was also evident when gating on IV label-negative parenchymal B cells (Figures S7F and S7G). EBI2−/− T cells did not have significant tumor homing defects (Figure 6F), showing that EBI2−/− lymphocytes were not globally deficient in tumor homing. However, the CD8 Tcm subset, which is enriched in EBI2 expression (Figure S4A), was modestly more EBI2-dependent in tumor homing than total CD8 T cells (Figures S7H and S7I). Overall, these data suggest that EBI2 promotes naive B cell entry into tumors from the blood.

### CCL19 plays a non-redundant role in lymphocyte recruitment to inflamed LNs

**Figure 7.**
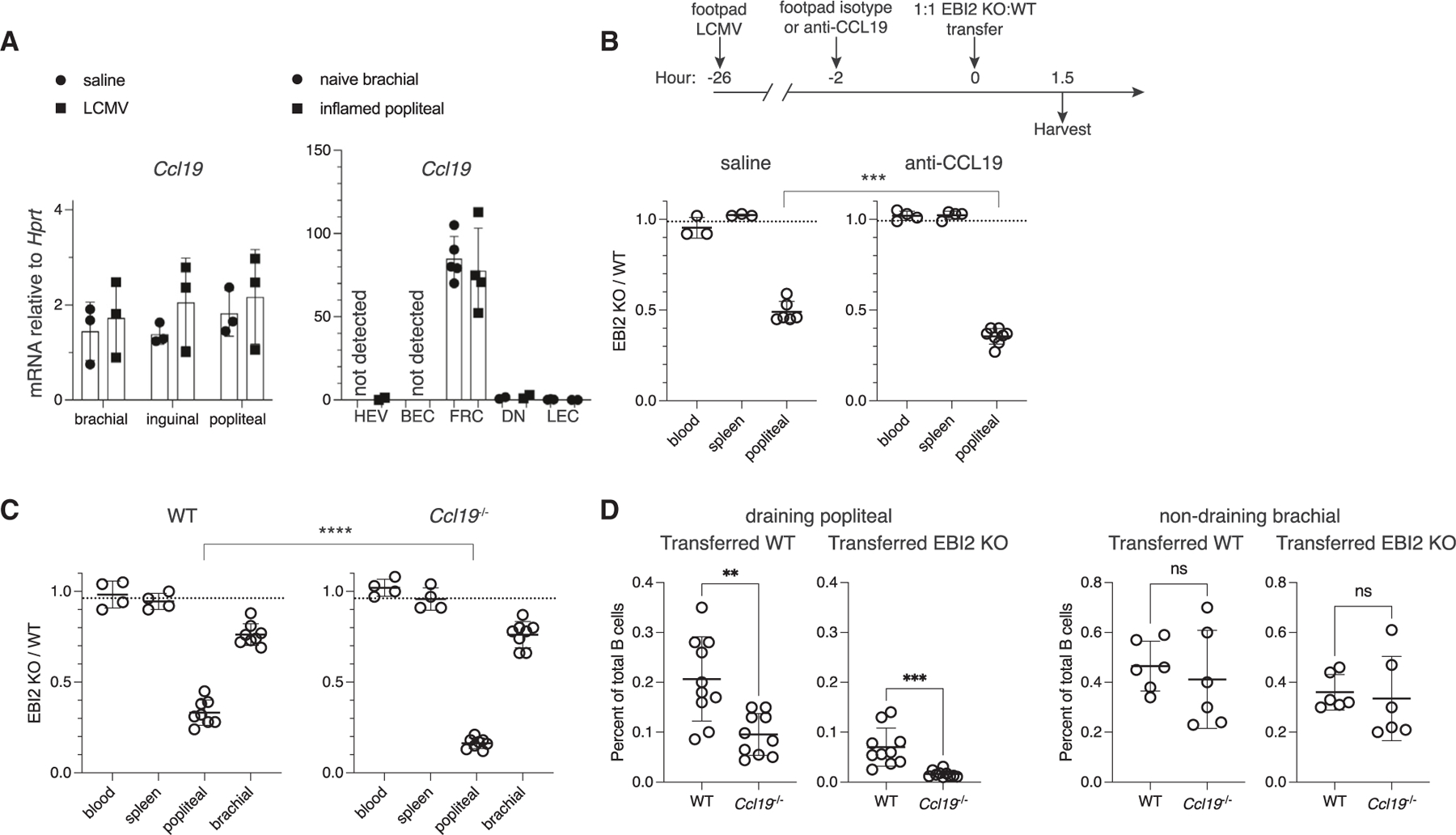
Inflammation reveals a role for CCL19 in naive lymphocyte homing that is non-redundant with CCL21 (A) RT-qPCR for *Cc19* in LNs (left) or sorted LN stroma (right) from saline or LCMV-infected mice. Each dot represents an LN pair from a mouse (left) or pooled sorted cells from 6 mice. (B) Schematic of experiment (top). Quantification of EBI2 KO:WT B cell ratios (bottom) after co-transfer. (C) Quantification of EBI2 KO:WT B cell ratios after co-transfer in WT or Ccl19−/− mice, post LCMV infection. The dashed line is the average input ratio from 3 experiments in (B) and (C). (D) Quantification of (C) for percent of total B cells that are transferred B cells in inflamed pLNs or naive bLNs of WT or Ccl19−/− mice. Each dot represents a mouse (blood, spleen) or individual LNs (popliteal, brachial) in (B)–(D). All statistical tests are two-tailed unpaired t tests. **p < 0.01; ***p < 0.001; ****p < 0.0001; ns, not significant (*p* > 0.05). Means ± standard deviation indicated in summary graphs. See also Figure S7.

While our experiments above indicated an important role for oxysterols and EBI2 in naive lymphocyte homing to inflamed LNs, mice lacking these factors still supported measurable lymphocyte recruitment. In our analysis of gene expression changes in inflamed LNs, we noted that mRNA and protein for the second CCR7 ligand, *Ccl19*, were sustained (Figures 7A and S3J). Moreover, CCR7−/− B cells showed similar magnitudes of homing defects to naive and inflamed pLNs despite the strong CCL21 modulation (Figures S7J and S7K). CCL21 repression could also impact the activity of CCL19 by reducing competition for extracellular matrix presentation sites in the T-zone and on HEVs. When we incubated pLN sections with CCL19-Fc or LFA3-Fc control reagent to test CCL19 binding activity, increased CCL19-Fc staining was detected in the T-zone and on HEVs of inflamed LNs, potentially indicating greater capacity of inflamed stroma to bind and present CCL19 (Figures S7L–S7N).

To test for a possible role of CCL19 in lymphocyte recruitment to inflamed LNs, we injected polyclonal CCL19 blocking antibody or isotype control into the footpads of mice that had been infected with LCMV 24 h prior (Figure 7B). 2 h later, 1:1 EBI2−/−: WT adoptive transfer was performed. Inflamed pLNs given anti-CCL19 were more dependent on EBI2 for recruiting transferred B cells (Figure 7B). This suggested that blocking CCL19 places further onus on EBI2 for inflamed LN entry and that CCL19 was no longer redundant with CCL21 in mediating lymphocyte recruitment into inflamed LNs.

Finally, we performed EBI2−/−:WT mixed transfers in CCL19−/− mice. At 24 h post-footpad infection with LCMV, naive lymphocyte entry to CCL19−/− inflamed pLNs was more dependent on EBI2 (Figures 7C and S7O). Moreover, WT B cell homing to inflamed LNs in CCL19−/− mice was 2-fold less efficient than in littermate controls, with even greater fold changes among transferred EBI2−/− B cells (Figure 7D). T cells showed similar trends (Figure S7P). This CCL19-dependent lymphocyte entry was specific to inflamed pLNs, as CCL19−/− non-draining bLNs displayed no homing defects, consistent with previous studies in naive mice (Figure 7D). Notably, EBI2−/− B cell homing to CCL19−/− inflamed pLNs was 0.017% of total B cells, whereas WT B cells were on average 0.2% of total B cells in WT pLNs, a 10-fold difference in homing efficiency (Figure 7D). Thus, the double loss of EBI2:oxysterol and CCR7:CCL19 chemoattraction renders lymphocytes severely impaired in inflamed LN entry.

## DISCUSSION

Our study demonstrates that the canonical chemoattractant receptor-ligand systems well-established for lymphocyte homeostatic trafficking to LNs are no longer the major contributors in the inflamed setting. The downregulation of CCL21 concomitant with retained CCL19 and upregulated Ch25h switches the signals naive lymphocytes use to access responding LNs from the blood.

The expression of Ch25h by HEVs and of Cyp7b1 by FRCs suggests a model where 7α,25-HC is made in a stepwise, transcellular manner involving production and release of 25-HC by HEVs, conversion to 7α,25-HC by FRCs, and then action on EBI2+ lymphocytes that are adherent in HEVs. This pattern of ligand generation may help ensure the existence of a decaying gradient from the parenchyma to the HEV lumen-facing endothelial surface. High expression of the 7α,25-HC catabolic enzyme HSD3B7 by HEVs compared with FRCs^50^ may also contribute to local gradient formation.

The factors leading to CCL21 downregulation in inflamed LNs appear complex. Investigation of CCL21 downregulation in the spleen at day 8 of LCMV infection identified a role for IFN-γ.^17^ However, we did not observe an effect of IFN-γ or IFNγR1 deficiency on acute CCL21 downregulation in LNs. In another study, CCL21 downmodulation in pLNs following *Salmonella* injection was suggested to involve stromal toll-like receptor 4 (TLR4) engagement,^18^ a mechanism unlikely to be active during LCMV infection. Further work on the cytokines or other potential signals, such as shear stress from lymphatic flow,^51^ regulating CCL21 expression will aid in elucidating the mechanism of CCL21 downregulation and its evolutionary significance.

Outside of the LNs, we show that EBI2 can promote naive B cell entry into MC38 tumors. In the tumor, expression of Ch25h by venules or myeloid cells may help with initiating anti-tumor responses, as early B cell entry may promote tertiary lymphoid structure formation.^52^ Given the sufficiency of IFN-α/β and IFN-γ to induce Ch25h, we suggest that oxysterol EBI2 ligands may be more readily produced within tumors and at sites of inflammation than CCL21 and thus may have an early role in lymphocyte recruitment to these sites. EBI2 contributes to effector T cell accumulation in the inflamed central nervous system^53^ possibly providing another example of EBI2-mediated recruitment from blood into inflamed tissue.

Our work adds a further function to an enigmatic DC subset. Selective depletion of LCs has shown a role for this cell type in promoting Th17 and Tfh responses to skin-associated pathogens and in regulating contact hypersensitivity responses.^54^ We show here that LCs upregulate Ch25h during viral infection and support EBI2-dependent naive lymphocyte entry to skin-draining LNs. Thus, in addition to shaping effector T cell responses, LCs influence the LN chemoattractant environment during inflammation.

Lastly, our work establishes a function for CCL19, which has previously been thought dispensable for lymphocyte positioning and homing in adult mice. CCL19 promotes splenic T-zone formation during development and contributes to naive T cell maintenance through a poorly understood mechanism.^14,55^ However, in adult mice at homeostasis, CCL21a KO leads to LN T cell homing defects, while CCL19 KO does not, thus showing that CCR7-dependent homing functions can be completely fulfilled by CCL21.^8,12,14^ This has been puzzling, given that genes encoding CCL19 and CCL21 are well conserved among vertebrate species and the expression of both ligands in LN T zones is conserved between rodents and humans.^1,8,12,13,36^ Our work establishes CCL19 can compensate for physiological CCL21 loss in promoting naive lymphocyte homing to LNs during infection. These findings are likely to extend to a range of conditions since CCL19 was maintained and CCL21 downregulated across multiple days in lymphoid tissues responding to *Salmonella*, MCMV, and herpes simplex virus 1 (HSV-1) infection.^16,18,22^ Further work will be needed to determine the importance of oxysterols and CCL19 in the adaptive immune response during chronic inflammation.

### Limitations of the study

A combination of video rate intravital and high-resolution microscopy will be needed to discern the precise steps during transendothelial migration where CCR7-CCL19 and EBI2-7α,25-HC act. While our finding that LCs contribute to lymphocyte-recruiting oxysterol production is supported by expression, BM chimera, and cell ablation studies, a more definitive test will involve conditional Ch25h deletion in LCs. While some LCs were observed to be adjacent to HEVs, most were T-zone localized, and we infer that oxysterols travel several cell diameters to engage HEV-associated lymphocytes. As future work investigates EBI2 action in LNs draining tissues other than skin, it will be of interest to assess oxysterol production by further myeloid cell types.

## STAR★METHODS

### RESOURCE AVAILABILITY

#### Lead contact

Requests for further information and resources should be directed to and will be fulfilled by the lead contact, Jason G. Cyster (jason.cyster@ucsf.edu).

#### Materials availability

This study did not generate new, unique reagents.

#### Data and code availability

- ATAC-seq data have been deposited at GEO: GSE281684 and are publicly available as of the date of publication.
- No new code was included in this study.
- Any additional information required to reanalyze the data reported in this paper is available from the lead contact upon request.

### EXPERIMENTAL MODEL AND SUBJECT DETAILS

#### Animals

C57BL/6J, BoyJ (CD45.1), and C57BL/6JxBoyJ (CD45.1 and CD45.2) mice were bred in an internal colony and 7–12 week-old mice of both sexes were used. EBI2−/−^59^ and Cyp7b1−/−^60^ mice backcrossed more than 11 and 5 generations, respectively, to C57BL/6J were from an internal colony. Ch25−/− and Ch25h^fl/fl^ mice were generated using CRISPR-based gene modification in C57BL/6J blastocytes.^61^ mTmG (Gt(ROSA)26Sor^tm4(ACTB-tdTomato,-EGFP)Luo^/J) reporter mice were a kind gift from Mark Looney (UCSF). Cdh5-CreERT2 (Tg(Cdh5-cre/ERT2)CIVE23Mlia) mice were a kind gift from Brian Black (UCSF). HuLang-DTR+ mice^46^ were a kind gift from Tiffany Scharschmidt (UCSF). Vav-iCre (Tg(Vav1-icre)A2Kio), Zbtb46-DTR+, Ifnar1−/−, Ifng−/−, and Ifngr1−/− mice were purchased from Jackson Laboratories. CCL19−/− mice^14^ that had been backcrossed to C57BL6/J 11x were cryo-recovered at Jackson Laboratories. CCR7−/− mice^7^ on a mixed B6 and 129 background were originally obtained from Martin Lipp (Max Delbrück Center for Molecular Medicine, Berlin).

To produce mixed chimeras, CD45.1 congenic C57BL/6 (Boy/J) mice were lethally X-ray irradiated with 900 cGy in split doses and reconstituted with 5 × 10^6^ BM cells of a mix of EBI2−/− CD45.2+ or CD45.2+ B6/J and CD45.1+CD45.2+ B6/J. Mice were analyzed 6–7 weeks later. To generate full chimeras, 5 × 10^6^ BM cells from Ch25h+/−, Ch25h−/−, Zbtb46-DTR+, or B6/J mice were transferred into lethally irradiated B6/J, Ch25h−/−, Vav-iCre+, Ch25h fl/fl, or Vav-iCre+ mTmG+ mice. Animals were housed in a specific pathogen-free environment in the Laboratory Animal Resource Center at the University of California, San Francisco, and all experiments conformed to ethical principles and guidelines that were approved by the Institutional Animal Care and Use Committee.

#### Cell lines

M12 B lymphoma cells,^58^ which were used for EBI2 ligand bioassays, were cultured in T-75 flasks in RPMI 1640 with 10% fetal bovine serum, 10 mM HEPES, 55 uM 2-mercaptoethanol, 2 mM glutamine, and 50 IU penicillin-streptomycin in a 37°C, 5% CO_2_ incubator. MC38 and bEnd.3 cells, which were used for subcutaneous tumor implantations and trans-endothelial migration assays, respectively, were seeded on 100 ¼m plates and cultured in DMEM with 10% fetal bovine serum, 10 mM HEPES, 2 mM glutamine, and 50 IU penicillin-streptomycin in a 37°C, 5% CO_2_ incubator.

### METHOD DETAILS

#### Mixed adoptive transfer

In co-transfer experiments, WT and EBI2−/− or CCR7−/− splenocytes were incubated at RT in ACK lysing buffer for 2 min and washed in PBS before being stained for 10 min at 37°C with 1uM CellTrace Violet (Thermo Fisher) or CellTracker Deep Red (Thermo Fisher). Cells were counted and mixed 50:50 before IV injection (10–15×10^6^ splenocytes / mouse in 100–200ul saline). Dyes were swapped between experiments to control for dye-labelling effects. To avoid effects due to CD45 locus linked genes,^69^ both WT and EBI2−/− donors were on a B6/J background. All transfers were for 90 min unless otherwise stated. For cleared LN imaging experiments, splenocytes were first negatively enriched for B cells via incubation with biotinylated anti-CD43 (clone eBioR2/60, Invitrogen), anti-CD3e (clone 145–2C11, Biolegend), and anti-CD11c (clone N418, Biolegend) followed by pull-down with EasySep streptavidin coated magnetic beads (Stemcell Technologies), before dye labelling. In some experiments, 2 μg / 200μl saline of PE-conjugated anti-mouse CD45.2 was injected IV per mouse 2 min before harvest to label only intravascular cells.

#### Infections and treatments

Mice were infected in the footpad with 1.2×10^5^ PFU of LCMV Armstrong in 40 ul saline. Mice were used at 24 hr after infection unless otherwise stated at indicated timepoints before mixed adoptive transfer. VSV was also delivered via footpad at a dose of 1×10^5^ PFU. For OVA administration along with LCMV, virus was diluted in saline with OVA such that each footpad received 10 ug of OVA along with 1.2×10^5^ PFU of LCMV Armstrong. For tail base injections, mice were shaved at the tail base and a single subcutaneous injection of 3×10^5^ PFU of LCMV Armstrong was administered. CFA emulsions (Sigma Aldrich) were prepared as previously described^70^ with a 1:1 ratio of saline:CFA and the thick white emulsions were tested for lack of dispersion on the surface of a saline solution. 30–40 μL of CFA was injected per footpad. For intranasal influenza infections, mice were anesthetized using isoflurane. When their breathing rate reached 1 breath per second, 500 PFU of PR8 H1N1 influenza virus in 40 uL of saline was administered to both nares. Mice were held upright for a minute to ensure little to no reflux was observed.

For mice requiring DT-mediated ablation before experiments, DT (Millipore) was diluted in saline for the appropriate dosages. Zbtb46-DTR+ chimeras and control chimeras received a single IV injection of 20 ng DT/g body weight. HuLang-DTR+ or WT mice received an IV dose of 30 ng DT/g body weight followed by a 30 ng dose of DT per footpad mixed with LCMV in saline to target LCs in the draining pLN during infection.

For blocking antibody or chemokine delivery experiments, 5 μg of polyclonal goat anti-CCL19 (R&D Systems) or anti-CCL21 (R&D Systems) or normal goat IgG control (R&D Systems) in saline were injected per footpad 2 hours prior to mixed adoptive transfer. For chemokine deliveries, 2.5 μg of CCL19 (R&D Systems) or CCL21b (Peprotech) in saline or saline alone were injected into contralateral footpads.

For mice requiring tamoxifen treatment, mice were injected IP daily for 5 days with 2 mg tamoxifen (Sigma) dissolved in corn oil or 90:10 sunflower oil:EtOH. Constant dosing with 10 mg tamoxifen spread across consecutive days was shown to maximize efficiency of Cdh5CreERT2-mediated recombination.^71^ Mice were rested for one week after tamoxifen treatment before experiments to allow for some of the tamoxifen and oil, which could affect cholesterol metabolism, to clear out.

#### Flow cytometry and cell sorting

LNs and spleen were processed via smashing through a 100 μm cell strainer in FACS buffer (PBS+2% NBCS+2mM EDTA). Spleens were incubated with ACK lysing buffer for 2 min before staining. For cell transfers, spleens were instead smashed in RPMI+10 mM HEPES to avoid EDTA chelation interfering with key homing proteins, such as integrins. Cells were only handled at RT or 37°C for dye labelling to avoid temperature shift-induced selectin shedding on cells used for short-term adoptive transfers. For mLNs and PP processing, samples were collected in complete RPMI (10% FCS, 10 mM HEPES, 2 mM glutamine, 55 μM 2-mercaptoethanol and 50 U penicillin/streptomycin) on ice and additionally washed with FACS buffer before staining. Blood was incubated twice with ACK lysing buffer for 5 min at RT and additionally washed with FACS buffer before staining. For experiments requiring staining and sorting of LN DCs and/or stromal cells, additional processing was required. For DCs and macrophages, LNs were collected in complete DMEM (10% FBS, 10 mM HEPES, 2 mM glutamine and 50 IU/L penicillin/streptomycin) and sliced open with 26-gauge needles to release lymphocytes and break open the LN capsule. Large pieces of LN were placed into DMEM digestion buffer containing 10 mM HEPES, 50 U penicillin/streptomycin, 2% FCS, 2 mg/mL collagenase IV (Worthington), and 80 ug/ml DNAse I (Sigma Aldrich). Supernatant containing mostly lymphocytes were collected in a separate tube. LN pieces were incubated at 37°C in digestion buffer for 30 min with mechanical agitation every 10 min by pipetting up and down with a P1000 pipette. LN pieces were then allowed to sediment for 1–2 min before collection of the supernatant containing largely DCs, macrophages, and lymphocytes. If stromal cells were not needed, the remaining LN pieces were smashed through a 100 μm cell strainer in FACS buffer. If stromal cells were desired, DMEM digestion buffer containing 10 mM HEPES, 50 U penicillin/streptomycin, 2% FCS, 3 mg/mL collagenase D (Sigma), and 40 ug/mL DNAse I were added to the remaining LN pieces. Samples were incubated for 5 min at 37°C, mechanically dissociated again with pipetting, followed by another 10 min incubation at 37°C. Samples were pipetted up and down again for 1–2 min. 5 mM EDTA was added to each sample to quench the digestion buffer, followed by 5 min of additional pipetting to ensure no solid material remains. Single cell suspensions were washed with FACS buffer before plating. For skin digestion and sorting, mouse footpad skin was peeled from the ankle toward the nails and then finely minced with scissors in 1 mL of skin digestion media (RPMI, 2% FCS, 10 mM HEPES, 1 mg/mL hyaluronidase, 50 ug/mL DNAse I, and 0.25 mg/mL liberase TM). Skin samples were incubated for 1 hour at 37°C with periodic agitation, followed by quenching with 800 uL of wash buffer (RPMI, 2% FCS, 10 mM HEPES, 5 mM EDTA). Samples were then smashed through 100 mm cell strainers in wash buffer. Samples were resuspended in FACS buffer for staining. For flow cytometry staining, cells were placed in a 96-well round bottom plate and washed with staining buffer (PBS containing 2% NBCS and 0.5 mM EDTA), and 30–40 μl of antibody cocktail was added to each sample and incubated for 20 min-45 min on ice. After incubation, cells were washed twice with staining buffer. Data were acquired using a BD LSR II flow cytometer or a Cytek Aurora. A BD FACSAria II was used to sort murine DCs or stromal cells with a 100 μm nozzle to minimize shear stress (post-sort purity >96%). Flow cytometry data were analyzed using Flowjo (v.10.6.2).

#### Transendothelial migration assay

bEnd.3 cells were grown in 10-cm tissue culture dishes in complete DMEM. To perform a lymphocyte in vitro transmigration assay, bEnd.3 cells were trypsinized and resuspended at 4–5×10^5^ / ml in complete DMEM. 24 well plate containing 0.6ml DMEM per well were prepared, transwells (5um pores, Corning) were inserted in the plates, and 100ul of bEnd.3 cell mix were gently pipetted on each transwell. bEnd.3 cells were also plated in a well without a transwell to estimate when the cells become a confluent monolayer. bEnd.3 cells were allowed to grow on transwells for 48 hrs. Before the migration assay, excess medium was removed, transwells were washed once in complete DMEM and placed in new 24-well plates containing indicated dilutions of migration stimuli in migration medium (RPMI+50 U penicillin/streptomycin+10mM HEPES+0.5% fatty acid-free BSA). 7α,25-HC (Cayman Chemical) stock solutions were 1mg/ml (2.39 mM) in EtOH, so nil migration stimuli contained an equivalent dilution of EtOH as 7α,25-HC migration stimuli to serve as vehicle control. WT or EBI2−/− splenocytes were diluted at 2×10^6^ / ml and 100ul of cell mix in migration medium were gently pipetted on bEnd.3 transwells. Cells were allowed to migrate for 3hr at 37°C. Harvested cells were centrifuged, washed, and resuspended in FACS buffer for flow cytometry analysis. To test integrin dependency, cells were inhibited with 20ug/ml of anti-αL and anti-α4 blocking antibody (BioXcell) for 15 min at 37°C before the assay. Data are plotted as % of input migration.

#### EBI2 ligand bioassay

For bioassays, LN extracts were prepared by weighing pooled naïve or inflamed pLNs and then mashing into migration media at a 1:100 mg:μL weight by volume ratio through a 70μm filter. ILNs were mashed at a 1:20 ratio instead. LN suspensions were then spun for 10 minutes at 300g to remove cells. Supernatants were spun again for 10 min at 3000g, and the supernatant from the second spin was used as extract. All extracts were further titrated in migration media for use in transwell migration assays. EBI2-transduced M12 cells were generated by spinfecting cells (2400 RPM, 2 hr at RT with no brake) with retrovirus encoding a MSCV-EBI2-IRES-GFP plasmid (virus produced using Platinum-E packaging cell line). Before bioassays, cells were grown to 70–80% confluency in T75 flasks, washed three times in migration media, and resensitized for 20 min at 37°C in migration media prior to use in migration assays. 1×10^6^ M12 cells were added to each transwell and allowed to migrate for 3 hr towards extracts. Data are shown as percent of input cells that migrated.

#### Tumor implantation and harvest

Mice were shaved on the flanks one day before tumor implantation and injected subcutaneously in the flank with 2×10^5^ MC38 tumor cells, which were grown in complete RPMI, or saline in the contralateral flank. Mice bearing day 16 tumors were euthanized, and blood was first collected via cardiac puncture, followed by lymphoid organs. Using surgical scissors, tumors were teased away from surrounding fat and underlying skin while minimizing bleeding from surrounding vessels. Nearby iLNs were dissected before tumor resection to avoid cross-contamination. Tumors were harvested into RPMI+10mM HEPES+2% NBCS on ice and then transferred to digestion buffer containing RPMI+10mM HEPES+2% NBCS+1mg/mL collagenase IV+100 ¼g/mL DNAse I (1 mg/mL of collagenase D was used instead of collagenase IV if CD62L staining was desired). Tumors were digested in 1ml digestion buffer / 0.2 g tumor and snipped into smaller fine pieces before incubation at 37°C, with periodic shaking, for 30 min. Wash buffer (RPMI+10mM HEPES+2% NBCS+5 mM EDTA) was added to obtain final concentration 2.5 mM EDTA to quench digestion buffer. Tumors were smashed through 100 μm cell strainers with wash buffer until only fine pieces of stromal cells remained on the cell strainer. Tumor cells suspensions were spun and washed in FACS buffer before plating for staining. 25 μl of CountBright Absolute Counting Beads (Life Tech) were added to each sample, and tumors were stained at a cell density of 3–4×10^4^ cells/μL of antibody cocktail for 30 min on ice. After incubation, cells were washed twice with FACS buffer.

#### Quantitative PCR

Total RNA from sorted LN DCs, stromal cells, or whole LNs was extracted using an Rneasy kit (Qiagen) and reverse-transcribed using M-MLV reverse transcriptase (Fisher). In some cases, the SuperScript^™^ III Reverse Transcriptase (Fisher) was used instead for low sorted cell number samples. qPCR was performed using Power SYBR Green (Life Tech) with an Applied Biosystems StepOnePlus instrument. Data were analyzed with the comparative threshold cycle C_t_ (2^−ΔΔCt^) method, using the housekeeping genes indicated in the figures. All primer set sequences used in this study can be found in Table S1.

#### ATAC-seq

ATAC-seq was performed according to a published Omni-ATAC protocol^72^ with minor modification. 5–8×10^3^ sorted LN FRCs were obtained from pooled pLNs or bLNs of 4–5 mice 24 hr after footpad LCMV infection. The cells were centrifuged at 500 r.c.f. for 5 min at 4°C in a fixed-angle centrifuge. Cell pellets were lysed and tagmented in one step by resuspending in 25 ul of tagmentation reaction mix containing 0.02% digitonin, 0.1% Tween-20, and 100 nM Tn5 transposase (^73^, provided by Emory Integrated Genomics Core) in tagmentation buffer (10 mM Tris-HCl, 5 mM MgCl2, and 10% dimethyl formamide). Transposition reactions were incubated at 37 °C for 60 min in a thermocycler. Reactions were cleaned up with Zymo DNA Clean and Concentrator 5 columns (Zymo Research). The remainder of the ATAC-seq library preparation was performed as described previously.^74^

#### ATAC-seq analysis

ATAC-seq reads from two biological replicates for each sample were mapped to the mouse genome (mm39 assembly) using Bowtie 1.3.1.^62^ In all cases, redundant reads were removed using FastUniq,^63^ and customized Python scripts were used to calculate the fragment length of each pair of uniquely mapped paired-end (PE) reads. Only one mapped read to each unique region of the genome that was less than 175 bp was kept and used in peak calling. Regions of open chromatin were identified by MACS (version 1.4.2)^64^ using a p-value threshold of 1×10^−5^. Only peaks detected in both replicates were used in downstream analysis. Peak intensities (“tags” column) were normalized as tags per 10 million reads (RP10M) in the original library. The annotation was carried out using PAPST^65^ and the heatmap creation utilized deeptools 3.5.4.^66^ Gene ontology and transcription factor regulation analysis on differentially accessible genes were performed through Metascape.^67^

#### Human LN single-cell RNA sequencing

Stromal cells from human mesenteric LN were isolated as previously described^75,76^ and processed for single-cell RNA sequencing using Chromium Single Cell 3′ (version 3) Library and Gel Bead Kit (10x Genomics) according to manufacturer guidelines. Libraries were sequenced on NextSeq500 (Illumina) at Stanford Functional Genomics Facility. Cell Ranger (v3.0, 10x Genomics) was used to align reads to the hg38 reference genome and perform quality control. The scran package was used to normalize raw count data and identify variable genes. Blood endothelial cell clusters were determined by maximal correlation with previously annotated lymph node blood endothelial cell datasets,^50^ and were visualized with Uniform Manifold Approximation and Projection (UMAP). To recover gene-gene relationships that are lost due to dropouts, gene expression data from log normalized count data was imputed using the MAGIC (Markov Affinity-based Graph Imputation of Cells) algorithm with optimized parameters (t = 2, k = 9, ka = 3) as previously described.^50^ Imputed data were used for visualization of single-cell gene expression in scatter plots.

#### Human skin single-cell RNA sequencing analysis

We sourced single-cell RNA sequencing data from,^48^ covering samples from human atopic dermatitis (AD) lesions and healthy control skin, stored in NCBI GEO under accession code GEO: GSE153760. Our analysis utilized R (version 4.2.1) and Seurat (version 4.3.0)^68^ focusing on AD5–8 skin biopsy samples. Quality control excluded cells with <200 or >4,000 genes detected, or >20% mitochondrial gene expression. After normalization, principal component analysis (PCA) identified 20 significant components. Dimensionality reduction and visualization were performed using RunUMAP, with cell subsets manually annotated following cluster marker identification by Seurat’s FindAllMarkers function. Clusters dominated by mitochondrial genes were removed. The endothelial cell cluster, consisting of 913 cells, was specifically analyzed using FeaturePlot for gene expression visualization.

#### RNA-scope

RNA in situ hybridization was performed using the RNAscope RED 2.5HD manual assay kit (Advanced Cell Diagnostics). The RNA-scope probes used were: Ch25h (NM_009890.1, targeting bp 115–1240) and Cyp7b1 (NM_007825.4, targeting bp 179–1182). Tissues were frozen in OCT and stored at −80°C. Within 24 hr of freezing, cryosections of 14 μm were cut and slides were dried at −20°C for 1 hr and at −80°C for 30 min. Slides were fixed for 15 min with ice-cold 4% paraformaldehyde and washed in 50%, 70%, and 100% ethanol for 5 min each. After drying for 5 min, slides were treated with hydrogen peroxide (from kit) for 8 min and protease IV (from kit) for 15 min. Probes were allowed to hybridize in a humidified chamber at 40°C for 3.5 hr. The following incubation times for the amplification steps were used: Amp 1, 40 min; Amp 2, 25 min; Amp 3, 40 min; Amp 4, 25 min; Amp 5, 40 min; Amp 6, 25 min. Slides were then developed with FastRed (from kit) for 20 minutes, washed in PBS, and counterstained for IgD using goat anti-mouse IgD (Cedarlane Laboratories) and HRP conjugated donkey anti-goat IgG (Jackson Immunoresearch).

#### Immunofluorescence microscopy

LNs were fixed in 1.6% paraformaldehyde in saline for 2 hours at 4°C, after which tissues were washed in saline and placed in 30% sucrose at 4°C overnight until LNs were dense enough to sink to the bottom of the sucrose solution. Tissues were washed, dried, and oriented in OCT to acquire both medulla and follicle views with each section of the LNs. Cryosections of 7μm were dried for 30 min and then blocked with blocking buffer containing PBS+5% NBCS+0.5% Tween-20+5ug/mL anti-CD16/32 (BioXCell) for 1 hr at RT. Sections were washed by incubating in staining buffer (PBS+1% NBCS+0.1% Tween-20+2.5ug/mL anti-CD16/32) for 5 min, followed by 2–3 hr incubation with primary antibody (1–5 μg/mL; 10 μg/mL for 4 hr for polyclonal goat-anti CCL19) cocktail at RT. Sections were washed and then incubated with secondary antibodies (5 μg/mL) or fluorophore-conjugated streptavidin (5 ug/ml) for 1–2 hr at RT. LFA3-human Fc and CCL19-human Fc generated as described^56^ were tested for tissue binding by following a similar protocol, and the reagent was titrated for optimal signal to background ratio on flow cytometry and imaging. PE-conjugated goat anti-human IgG F(ab’)_2_ preabsorbed on 2% mouse and rat serum was used for Fc fusion detection, followed by staining for lineage markers such as IgD, CD4, or PNAd. Sections were washed in staining buffer and mounted in Fluoromount-G with no.1.5 coverslips. Images were captured with an inverted Zeiss AxioObserver Z1 microscope. Images were analyzed in Fiji and brightness and contrast settings were set to the same level for all images from the same experiment to allow quantitative comparison of intensity levels by eye.

#### LN clearing and two-photon imaging

LNs were harvested from LCMV footpad infected mTmG mice 90 min after mixed adoptive transfer of 5–7×10^5^ purified B cells and 2 min after a 2ug anti-CD45-AF488 (clone 104, Biolegend) IV label to highlight HEVs. LNs were fixed in 1.6% PFA at 4°C for 2 hours. LNs were then washed in PBS and fat was carefully cleaned off with sharp forceps to ensure minimal obstruction during imaging. LNs were placed in clearing solution made based on the Ce3D clearing protocol.^77^ The clearing solution contained 86% w/g Histodenz dissolved in 40% v/v N-methylacetamide in PBS with 0.1% v/v Triton X-100 and 0.5% v/v 1-thioglycerol. LNs were rotated in clearing solution for 24 hours, after which the LNs were transferred to fresh clearing solution for another 24-hour incubation. LNs were mounted in the same clearing solution to match the refractive index of the sample and surrounding medium. A no. 1.5 coverslip was placed on top of a thin layer of vacuum grease on coverglass to create a sealed chamber for the LN in clearing solution. Imaging was conducted using a STELLARIS 8 multiphoton microscope (Leica) equipped with two Chameleon lasers and water dipping 25x two-photon objective. Two-photon imaging depth reached to ~500–600 um below the LN capsule, with emission light scattering limiting imaging depth for shorter wavelength emission dyes, such as CellTrace Violet. Z-stacks of about 400 um in depth were obtained at 2 um steps with power ramping of about 0.1% laser power increase per um to normalize image intensities across z. Images were acquired with 1040 nm and 800 nm excitation with line average 6 and 20% overlap between tiles.

#### 3D image processing and quantification

The 3D, tiled z-stack images of cleared LNs were first stitched in Leica LAS X software. Stacks were spectrally unmixed in Leica LAS X to reduce crosstalk between channels and aid in downstream cell and HEV identification steps. For spectral unmixing, regions of single colors were selected to measure the profile of each dye, which were used to generate the unmixing matrix. This enhanced the tdTomato vasculature outlines around CD45 IV label within HEVs. Analysis of transferred B cell positions were conducted with Imaris V9.3.1 (Bitplane). First, surfaces were made on high intensity objects that were bright in all channels and those autofluorescent objects were masked. Next, one to two ~300 μm x 300 μm x 300 μm regions centered on an HEV structure were cropped out of each LN 3D stack to facilitate analysis. HEVs were manually contoured in Imaris using a combination of the CD45 IV label and surrounding tdTomato signal. CTV and DR labelled B cells were identified in Imaris using automatic spot detection (XY cell diameter estimate ~5 μm with ~10 μm z-diameter to account for axial length of point spread function). Various filters were applied to exclude low signal to noise quality objects followed by manual editing to add missed cells, which were usually near the bottom of the z-stack with lower signal, or delete any residual autofluorescent objects that were not bona fide dye-labelled transferred cells. The shortest distance to HEV was calculated for each transferred B cell and cells <1 um to the nearest HEV were labelled as within HEV. 1 um was used instead of 0 um because many cells were observed within the HEV lining, consistent with previous observations of lymphocytes residing in HEV ‘pockets’ during transmigration.^78^

### QUANTIFICATION AND STATISTICAL ANALYSIS

Prism software (GraphPad 9.0.1) was used for all statistical analyses. All statistical tests are two-tailed unpaired t-tests, unless noted in the figure legend that a paired t-test was used to compare internally controlled groups. *p < 0.05; **p < 0.01; ***p < 0.001; ****p < 0.0001; ns, not significant (*p* > 0.05). In summary graphs, points indicate individual samples, and horizontal lines are means unless otherwise noted. All error bars are standard deviation.

## Supplementary Material

MMC2

MMC1

MMC3

## Figures and Tables

**Table:** KEY RESOURCES TABLE

REAGENT or RESOURCE	SOURCE	IDENTIFIER
Antibodies		

Goat Anti-Mouse Ccl21 / 6ckine Polyclonal antibody, Biotin Conjugated	R and D Systems	Cat# BAF457; RRID:AB_2072082
Alexa Fluor(R) 647 anti-mouse/human PNAd	BioLegend	Cat# 120808; RRID:AB_2783060
PE anti-mouse IgD	BioLegend	Cat# 405706; RRID:AB_315028
Brilliant Violet 510(TM) anti-mouse/human CD45R/B220	BioLegend	Cat# 103248; RRID:AB_2650679
Alexa Fluor(R) 488 anti-mouse CD45	BioLegend	Cat# 103121; RRID:AB_493532
Brilliant Violet 605(TM) anti-mouse CD19	BioLegend	Cat# 115540; RRID:AB_2563067
Alexa Fluor(R) 488 anti-mouse CD31	BioLegend	Cat# 102514; RRID:AB_2161031
Goat Anti-Mouse Ccl19 / mip-3 beta Polyclonal antibody	R and D Systems	Cat# AF880; RRID:AB_2071545
Normal Goat IgG Control	R and D Systems	Cat# AB-108-C; RRID:AB_354267
Alexa Fluor(R) 700 anti-mouse CD45.1	BioLegend	Cat# 110724; RRID:AB_493733
Brilliant Violet 421(TM) anti-mouse CD45.2	BioLegend	Cat# 109832; RRID:AB_2565511
Brilliant Violet 785(TM) anti-mouse/human CD44	BioLegend	Cat# 103059; RRID:AB_2571953
APC/Cyanine7 anti-mouse TCR Vα2	BioLegend	Cat# 127818; RRID:AB_10682897
PE anti-mouse TCR Vβ5.1, 5.2	BioLegend	Cat# 139503; RRID:AB_10612761
PerCP-Cyanine5.5 Anti-Mouse TCR beta (H57–597)	Tonbo Biosciences	Cat# 65–5961; RRID:AB_2621911
PE-Cyanine7 Anti-Mouse CD11c (N418)	Tonbo Biosciences	Cat# 60–0114; RRID:AB_2621837
PerCP/Cyanine5.5 anti-mouse I-Ab	BioLegend	Cat# 116416; RRID:AB_1953309
Brilliant Violet 711(TM) anti-mouse CD326 (Ep-CAM)	BioLegend	Cat# 118233; RRID:AB_2632775
Brilliant Violet 785(TM) anti-mouse/human CD11b	BioLegend	Cat# 101243; RRID:AB_2561373
APC anti-mouse/human CD207 (Langerin)	BioLegend	Cat# 144205; RRID:AB_2561997
Brilliant Violet 711(TM) anti-mouse CD4	BioLegend	Cat# 100447; RRID:AB_2564586
PE/Cyanine7 anti-mouse Podoplanin	BioLegend	Cat# 127412; RRID:AB_10613648
Goat anti-mouse IgD	Novus Biologicals	Cat# NBP2–69334; RRID:AB_3354390
PE anti-mouse CD45	BioLegend	Cat# 103106; RRID:AB_312971
PerCP/Cyanine5.5 anti-mouse CD8a	BioLegend	Cat# 100734; RRID:AB_2075238
InVivoMab anti-mouse/human VLA-4 (CD49d)	Bio X Cell	Cat# BE0071; RRID:AB_1107657
InVivoMab anti-mouse LFA-1α (CD11a)	Bio X Cell	Cat# BE0006; RRID:AB_1107578
Brilliant Violet 510(TM) anti-mouse CD62L	BioLegend	Cat# 104441; RRID:AB_2561537
PE/Cyanine7 anti-mouse CD279 (PD-1)	BioLegend	Cat# 109110; RRID:AB_572017
PE anti-mouse CD366 (Tim-3)	BioLegend	Cat# 119703; RRID:AB_345377
APC anti-mouse Ly108	BioLegend	Cat# 134610; RRID:AB_2728155
FITC anti-mouse CD169 (Siglec-1)	BioLegend	Cat# 142405; RRID:AB_2563106
APC/Cyanine7 anti-mouse F4/80	BioLegend	Cat# 123118; RRID:AB_893477
Alexa Fluor^®^ 647 anti-mouse CD64 (FcγRI)	BioLegend	Cat# 139322; RRID:AB_2566561
PE/Cyanine7 anti-mouse CD69	BioLegend	Cat# 104512; RRID:AB_493564
Brilliant Violet 605(TM) anti-mouse CD103	BioLegend	Cat# 121433; RRID:AB_2629724
Brilliant Violet 650(TM) anti-mouse IgD	BioLegend	Cat# 405721; RRID:AB_2562731
Alexa Fluor^®^ 700 anti-mouse CD3ε	BioLegend	Cat# 152316; RRID:AB_2632713
Biotin-SP-AffiniPure Goat Anti-Human IgG, Fc_ Fragment Specific	Jackson ImmunoResearch Labs	Cat# 109–065-098; RRID:AB_2337630
CD43 Monoclonal Antibody (eBioR2/60), Biotin, eBioscience	Thermo Fisher Scientific	Cat# 13–0431-85; RRID:AB_466440
Biotin anti-mouse CD3ε	BioLegend	Cat# 100304; RRID:AB_312669
Biotin anti-mouse CD11c	BioLegend	Cat# 117304; RRID:AB_313773
Peroxidase-AffiniPure Donkey Anti-Goat IgG (H+L)	Jackson ImmunoResearch Labs	Cat# 705–035-147; RRID:AB_2313587

Bacterial and virus strains		

LCMV Armstrong	University of California San Francisco, Nadia Roan	N/A
VSV-GFP Indiana	The Ohio State University, Glen Barber	N/A
Influenza A virus (H1N1) A/PR/8/34	University of California San Francisco, Mark Looney	N/A

Chemicals, peptides, and recombinant proteins		

Recombinant Mouse CCL19/MiP-3 beta Protein	R and D Systems	Cat# 440-M3/CF
Mouse CCL21 (6Ckine) Recombinant Protein	PeproTech	Cat# 250–13-20UG
CellTracker Deep Red Dye	Life Technologies	Cat# C34565
Freund′s Adjuvant, Complete	Sigma-Aldrich	Cat# F5881
Diphtheria Toxin, Unnicked	Sigma-Aldrich	Cat# 322326
Tamoxifen	Sigma-Aldrich	Cat# T5648
Type 4 collagenase, from Clostridium histolyticum	Worthington Biochemical	Cat# LS004188
Collagenase D, from Clostridium histolyticum	Roche	Cat# 11088866001
Deoxyribonuclease I from bovine pancreas	Sigma-Aldrich	Cat# DN25
Hyaluronidase from bovine testes	Sigma-Aldrich	Cat# H3506
Liberase TM Research Grade	Roche	Cat# LIBTM-RO
7α,25-dihydroxy Cholesterol	Cayman Chemical	Cat# 11032
Human CXCL12 (SDF-1a) Recombinant Protein	PeproTech	Cat# 300–28A
M-MLV Reverse Transcriptase	Invitrogen	Cat# 28025013
SuperScript^™^ III Reverse Transcriptase	Invitrogen	Cat# 18080044
Tn5 transposase	Emory Integrated Genomics Core	N/A
LFA3-human Fcγ1	In-house, Hargreaves et al.^56^	N/A
CCL19-human Fcγ1	In-house, Hargreaves et al.^56^	N/A
Histodenz	Sigma-Aldrich	Cat# D2158
N-Methylacetamide	Sigma-Aldrich	Cat# M26305
1-Thioglycerol	Sigma-Aldrich	Cat# M1753
CellTrace Violet	Invitrogen	Cat# C34557

Critical commercial assays		

RNeasy Mini Kit	Qiagen	Cat# 74004
DNA Clean & Concentrator-5	Zymo Research	Cat# D4003
Chromium Next GEM Single Cell 3’ GEM, Library & Gel Bead Kit v3.1	10x Genomics	Cat# PN-1000121
RNAscope^™^ 2.5 HD Reagent Kit-RED	Advanced Cell Diagnostics	Cat# 322350
EasySep Streptavidin RapidSpheres	StemCell Technologies	Cat# 50001
CountBright^™^ Absolute Counting Beads	Invitrogen	Cat# C36950
*Power* SYBR^™^ Green PCR Master Mix	Applied Biosystems	Cat# 4367660

Deposited data		

ATAC-seq of murine lymph node naive and inflamed fibroblastic reticular cells	This paper	GEO: GSE281684
scRNA-seq of epidermal and immune cells from human atopic dermatitis and healthy control skin	Rojahn et al.^48^	GEO: GSE153760
scRNA-seq of tumor-associated high endothelial cells	Asrir et al.^47^	GEO: GSE154898
ATAC-seq of lymph node reticular and CD34+ stromal cells	Pezoldt et al.^57^	GEO: GSE172526
ATAC-seq on splenic naive CD4^+^ T cells	Shih et al.^33^	GEO: GSE77695

Experimental models: Cell lines		

MC38	University of California San Francisco, Matthew F. Krummel	RRID:CVCL_B288
bEnd.3	ATCC	RRID:CVCL_0170
M12	Kim et al.^58^	RRID:CVCL_IU99

Experimental models: Organisms/strains		

Mouse: C57BL/6	Jackson Laboratories	RRID:IMSR_JAX:000664
Mouse: B6 CD45.1	Jackson Laboratories	RRID:IMSR_JAX:002014
Mouse: Ebi2−/−	Pereira et al.^59^	N/A
Mouse: Cyp7b1−/−	Li-Hawkins et al.^60^	N/A
Mouse: Ch25h−/−	Frascoli et al.^61^	N/A
Mouse: Ch25h fl/fl	Frascoli et al.^61^	N/A
Mouse: mTmG	University of California, San Francisco, Mark Looney	RRID:IMSR_JAX:007676
Mouse: Cdh5-CreERT2	University of California, San Francisco, Brian Black	MGI:3700149
Mouse: HuLang-DTR^+^	Bobr et al.^46^	N/A
Mouse: Vav-iCre	Jackson Laboratories	RRID:IMSR_JAX:008610
Mouse: zDC-DTR	Jackson Laboratories	RRID:IMSR_JAX:019506
Mouse: Ifnar1−/−	Jackson Laboratories	RRID:IMSR_JAX:028288
Mouse: Ifng−/−	Jackson Laboratories	RRID:IMSR_JAX:002287
Mouse: Ifngr1−/−	Jackson Laboratories	RRID:IMSR_JAX:003288
Mouse: CCL19−/−	Link et al.^14^	RRID:IMSR_JAX:012851
Mouse: CCR7−/−	Forster et al.^7^	N/A

Oligonucleotides		

RT-qPCR primers	Integrated DNA Technologies	see Table S1
RNAscope Probe- Mm-Ch25h	Advanced Cell Diagnostics	Cat# 424561; Accession# NM_009890.1
RNAscope Probe- Mm-Cyp7b1	Advanced Cell Diagnostics	Cat# 471001; Accession# NM_007825.4

Recombinant DNA		

MSCV-EBI2-IRES-GFP plasmid	In-house, Pereira et al.^59^	N/A

Software and algorithms		

FlowJo	TreeStar	https://www.flowjo.com/solutions/flowjo
GraphPad Prism	GraphPad	https://www.graphpad.com/
Imaris 9.3.1	Oxford Instruments	https://imaris.oxinst.com
Bowtie 1.3.1	Langmead et al.^62^	https://github.com/BenLangmead/bowtie/releases
FastUniq	Xu et al.^63^	https://anaconda.org/bioconda/fastuniq
MACS 1.4.2	Zhang et al.^64^	https://bioweb.pasteur.fr/packages/pack@macs@1.4.2
PAPST	Bible et al.^65^	https://github.com/paulbible/papst
deepTools 3.5.4	Ramirez et al.^66^	https://deeptools.readthedocs.io/en/develop/
Metascape	Zhou et al.^67^	https://metascape.org
CellRanger 3.0	10X Genomics	https://www.10xgenomics.com/support/software/cell-ranger
Seurat 4.3.0	Hao et al.^68^	https://satijalab.org/seurat/

## Highlights

- Inflamed LNs switch CCR7 ligand mediating lymphocyte recruitment from CCL21 to CCL19
- EBI2 and oxysterol ligand 7α,25-HC are required for lymphocyte homing to inflamed LNs
- Endothelium, fibroblasts, and Langerhans cells cooperate in oxysterol production
- Ch25h is in inflamed nonlymphoid endothelium, and EBI2 promotes B cell homing to tumors
